# Is GDF15 a Feasible Biomarker in Sepsis?

**DOI:** 10.3390/diagnostics15172224

**Published:** 2025-09-02

**Authors:** Ertugrul Yigit, Mehmet Akif Simsek, Merve Huner Yigit, Gorkem Akca, Berat Sonmez, Hakki Uzun

**Affiliations:** 1 Department of Medical Biochemistry, Faculty of Medicine, Karadeniz Technical University, Trabzon 61080, Türkiye; ertugrulyigit@ktu.edu.tr; 2 Department of Urology, Faculty of Medicine, Recep Tayyip Erdogan University, Rize 53000, Türkiye; mehmetakifsimsek24@gmail.com (M.A.S.); gorkem.akca@erdogan.edu.tr (G.A.); 3 Department of Medical Biochemistry, Faculty of Medicine, Recep Tayyip Erdogan University, Rize 53000, Türkiye; merve.huner@erdogan.edu.tr; 4 Department of Urology, Bingöl State Hospital, Bingöl 12000, Türkiye; drberatsonmez@gmail.com

**Keywords:** CRP, GDF15, sepsis, renal dysfunction, procalcitonin, presepsin

## Abstract

**Background/Objectives**: Sepsis is a high-mortality syndrome characterized by organ dysfunction resulting from a dysregulated host response to infection. This study aimed to evaluate the potential of growth differentiation factor 15 (GDF15), a stress-inducible cytokine, as a biomarker in patients diagnosed with urosepsis. **Methods**: A total of 13 patients diagnosed with urosepsis, based on an increase of ≥2 points in the Sequential Organ Failure Assessment (SOFA) score and positive urine culture, were included in the study. Daily blood samples were collected from patients for 10 days, and serum levels of GDF15, procalcitonin (PCT), and presepsin (P-SEP) were measured by ELISA. C-reactive protein (CRP), blood urea nitrogen (BUN), serum creatinine, estimated glomerular filtration rate (eGFR), hemoglobin, and neutrophil, lymphocyte, and platelet counts were determined using autoanalyzers. Temporal changes were analyzed using the Friedman test, and correlations were analyzed using Spearman’s test. **Results**: GDF15 levels began to decrease from Day 3, with a significant decline observed from Day 7 compared to Day 1 (*p* < 0.001). Similar decreasing trends were observed in CRP and PCT levels, whereas presepsin levels did not exhibit significant changes. Significant positive correlations were identified between GDF15 and CRP (r = 0.65, *p* = 0.015), BUN (r = 0.57, *p* = 0.041), and creatinine (r = 0.62, *p* = 0.024), and a significant negative correlation was observed with eGFR (r = −0.62, *p* = 0.024). No significant correlation was found between GDF15 and presepsin (*p* > 0.05). **Conclusions**: GDF15 is a biomarker sensitive to the resolution phase of inflammation and organ dysfunction in sepsis, demonstrating significant temporal changes. It holds potential as an indicator for monitoring clinical progression and assessing prognosis.

## 1. Introduction

Sepsis is a complex syndrome characterized by life-threatening organ dysfunction resulting from a dysregulated host response to infection [1]. The incidence of sepsis and septic shock has continuously increased, reaching approximately 49 million cases of sepsis and 11 million sepsis-related deaths worldwide in 2017 [2]. Urosepsis refers to sepsis caused by urinary tract infections (UTIs), including cystitis or lower urinary tract and bladder infections, as well as pyelonephritis or upper urinary tract and kidney infections [3]. UTIs are among the most common causes of sepsis, estimated to account for 20% to 40% of all sepsis cases [4].

Early identification and prediction of prognosis in sepsis are critically important for successful clinical management. In this context, various biomarkers, particularly C-reactive protein (CRP), procalcitonin (PCT), and presepsin (P-SEP), are utilized clinically for diagnosis and monitoring [5,6]. CRP is a classical acute-phase reactant synthesized by hepatocytes in response to cytokines such as IL-6 and increases in conditions like infection and sepsis. It serves as a general marker of systemic inflammation [7]. PCT, a more specific biomarker for bacterial infections, rapidly increases shortly after the onset of disease and is especially helpful in evaluating antibiotic therapy [8]. Presepsin, resulting from the cleavage of the CD14 receptor on monocytes, is identified as an early-phase response marker in sepsis [5,9].

CRP and PCT are widely used biomarkers for diagnosing and monitoring sepsis; however, both biomarkers have limited specificity. This limitation primarily stems from their elevated levels in non-infectious inflammatory conditions (such as trauma, surgery, and thermal injury) [10]. Although CRP is a classical acute-phase reactant synthesized in hepatocytes via IL-6, it is elevated not only in infections but also in malignancies, autoimmune diseases, and trauma. Similarly, while PCT is more specific to bacterial infections, its levels also increase in certain non-infectious clinical conditions, reducing diagnostic accuracy. Both biomarkers can indicate inflammation due to disease, but fall short in identifying a specific source of infection or sepsis subtype. Thus, given the complex immune response structure of sepsis, it is emphasized that a multi-biomarker approach may be more beneficial than relying on a single ideal biomarker [10,11].

In recent years, growth differentiation factor 15 (GDF15), a member of the Transforming Growth Factor-beta (TGF-β) family, has emerged as an intriguing molecule distinct from classical inflammatory markers. GDF15 levels rise in conditions involving oxidative stress, tissue damage, inflammation, and mitochondrial dysfunction [12,13]. Studies have shown that GDF15 is a significant biomarker in inflammation-related diseases such as cardiovascular diseases and cancer [14]. Additionally, the potential role of GDF15 as a diagnostic and prognostic marker in critical clinical conditions, such as sepsis, which pose significant diagnostic and therapeutic challenges, remains under investigation [15,16]. Unlike classical biomarkers, GDF15 reflects the organism’s adaptive response to systemic inflammation, tissue injury, and metabolic stress, suggesting its utility as a complementary biomarker in sepsis. Its weak correlations with classical inflammatory markers further indicate that GDF15 represents a distinct biological process in sepsis pathophysiology [16].

This study aims to compare the temporal changes in GDF15 levels with those of classical inflammatory biomarkers, such as CRP, PCT, and P-SEP, in septic patients, exploring its potential role as a biomarker for clinical monitoring in sepsis. Thus, the potential of GDF15 as a complementary biomarker in sepsis pathophysiology is investigated.

## 2. Materials and Methods

### 2.1. Ethical Approval, Study Design, and Patient Selection

This study was approved by the Clinical Research Ethics Committee of the Faculty of Medicine, Recep Tayyip Erdoğan University (Decision No: 2024/177; Approval Date: 18 July 2024). All procedures were conducted following the Declaration of Helsinki and the provisions of relevant national legislation [17]. This prospective observational study was conducted between September and December 2024 in adult patients (aged 49 to 86 years) evaluated in the emergency department and diagnosed with urosepsis secondary to acute pyelonephritis, who were subsequently hospitalized, treated, and monitored in the urology department for 10 days with antimicrobial therapy initiated according to the recommendations of the infectious diseases clinic. No additional infectious foci were identified, and all patients were diagnosed according to the Sepsis-3 criteria. The diagnosis of sepsis was made by relevant specialist physicians based on the presence of infection accompanied by a systemic inflammatory response and acute organ dysfunction, as determined by clinical and laboratory findings [18]. Two urologists followed all patients. A total of 13 patients with a positive urine culture at diagnosis (with or without concomitant positive blood culture), in whom GDF15, CRP, PCT, and presepsin could be monitored daily for 10 days, were included in the study. Written informed consent was obtained from all participants or their first-degree relatives. During the same period, renal function parameters and routine hematological indicators were also monitored. All demographic characteristics, detailed medical histories, physical examination findings, microbiological culture results obtained before treatment initiation, laboratory parameters, and clinical follow-up data of the patients were recorded in full. The microorganisms isolated from the study population included those commonly encountered in sepsis, such as *Escherichia coli*, *Klebsiella pneumoniae*, *Pseudomonas aeruginosa*, *Serratia marcescens*, *Enterococcus* spp., *Staphylococcus* spp., *Streptococcus agalactiae*, *Proteus mirabilis*, and *Candida* spp.

### 2.2. Exclusion Criteria and Comorbidity Assessment

The majority of patients included in the study had common chronic diseases associated with sepsis, including hypertension, diabetes mellitus, coronary artery disease, chronic kidney disease, history of kidney stones, COPD, benign prostatic hyperplasia, and dyslipidemia. These comorbidities were intentionally not excluded from the study; instead, they were included to reflect the real-life clinical profiles of the patients. This approach aimed to enhance the clinical relevance and generalizability of the biomarker data obtained. However, certain specific clinical conditions anticipated to affect GDF15 levels directly were set as exclusion criteria. Accordingly, patients with active malignancies, those receiving immunosuppressive or chemotherapeutic treatments, individuals with a history of autoimmune diseases, and patients diagnosed with severe liver disease were excluded from the study. Patients presenting with clinical signs or symptoms suggestive of non-urinary tract infections were excluded from the study.

### 2.3. Blood Sample Collection and Measurement of Biochemical Parameters

Peripheral venous blood samples were collected from patients every morning at the same time for 10 days starting from the first day of admission, to align with routine sepsis monitoring (CRP, leukocyte count, PCT) and to capture temporal biomarker kinetics while minimizing diurnal variation. Serum samples were transferred to disposable tubes following centrifugation and stored at −80 °C, thawed only once immediately before analysis. All measurements were evaluated using the double-blind method. GDF15 levels were measured using the R&D Systems DGD150 ELISA kit (Minneapolis, MN, USA) according to the manufacturer’s protocol. PCT levels were analyzed with the Elabscience E-EL-H1492 ELISA kit (Elabscience, Wuhan, China). Presepsin levels were assessed using the BT-Lab E3754Hu ELISA kit (BT-Lab Shanghai, China). All biomarker analyses were performed according to the manufacturer’s recommendations regarding sample volume, incubation duration, and absorbance wavelengths. CRP, blood urea nitrogen (BUN), and serum creatinine levels were measured using the Beckman Coulter AU5800 fully automated biochemistry analyzer (Brea, CA, USA). Estimated glomerular filtration rate (eGFR) was calculated using the modified MDRD formula based on serum creatinine values. Complete blood count parameters, including white blood cells (WBC), neutrophils (Neu), lymphocytes (Lym), hemoglobin (Hb), and platelets (Plt), were measured using the Mindray BC-6000 fully automated hematology analyzer (Shenzhen, China). The neutrophil-to-lymphocyte ratio (NLR) was calculated from these data and used as a supportive parameter for dynamic monitoring of inflammation.

### 2.4. Statistical Analysis

All statistical analyses were performed using OriginPro 2025 (OriginLab Corporation, Northampton, MA, USA). Continuous variables are presented as median (interquartile range) unless otherwise stated. Temporal changes in biomarker levels across the 10-day follow-up were evaluated using the Friedman test for repeated measures. Where overall significance was detected, Dunn’s post hoc test was applied for pairwise comparisons between each time point and Day 1. Statistically significant differences were defined as *p* < 0.05. In line graphs, an asterisk (*) indicates a statistically significant difference compared to Day 1 (*p* < 0.05). Correlation analyses between GDF15 and other inflammatory or renal biomarkers were performed using Spearman’s rank correlation coefficient. Pairwise associations were visualized in a correlation heat map, where a diverging color scale represents negative to positive Spearman’s ρ (blue to red), and color intensity reflects the magnitude of the coefficient. All statistical tests were two-sided.

## 3. Results

Detailed demographic, clinical, and biochemical data of the patients included in this study are presented in the Supplementary Materials section (see Supplementary Figures S1–S13).

### 3.1. Temporal Changes in Inflammatory Biomarkers

**Figure 1:**
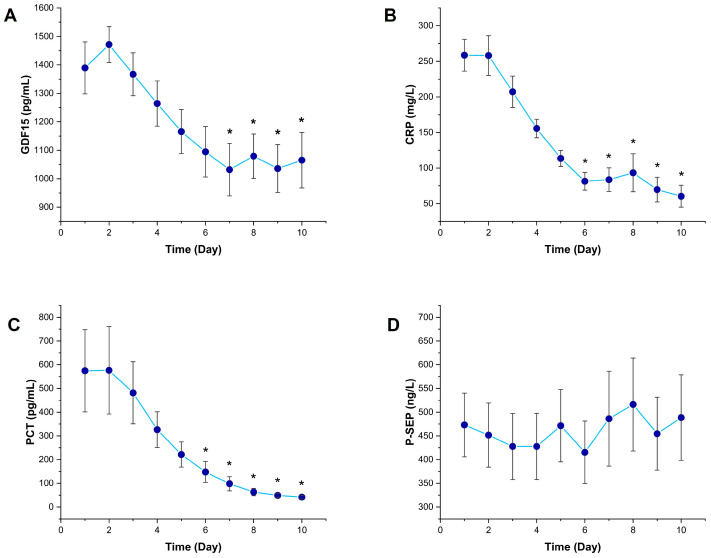
Temporal changes in inflammatory biomarkers in sepsis patients. Line graphs showing the 10-day trends of (**A**) growth differentiation factor 15, (**B**) C-reactive protein, (**C**) procalcitonin, and (**D**) presepsin levels in septic patients. Data are presented as mean and SD. * *p* < 0.05 vs. Day 1 by Friedman test followed by Dunn’s multiple comparisons post hoc test.

Among all inflammatory biomarkers, GDF15 exhibited a distinct temporal change pattern. During the 10-day follow-up period, GDF15 levels began to decrease after Day 3, and this decline reached statistical significance from Day 7 onward (*p* < 0.001), with levels being significantly lower compared to Day 1 starting from Day 7 (*p* < 0.05) (Figure 1A). This finding suggests that GDF15 may play a compensatory or anti-inflammatory role in the later phases of sepsis.

CRP levels began to decline from Day 2 and showed a statistically significant decrease compared to Day 1 from Day 6 onward (*p* < 0.05) (Figure 1B). PCT levels also showed a marked reduction starting from Day 2, and significantly lower values were observed compared to Day 1 during the period from Day 6 to Day 10 (*p* < 0.05) (Figure 1C). CRP and PCT decreased earlier, whereas GDF15 exhibited the slowest decline.

In contrast, no statistically significant changes were observed in presepsin levels throughout the 10-day follow-up period (*p* > 0.05) (Figure 1D).

**Figure 2:**
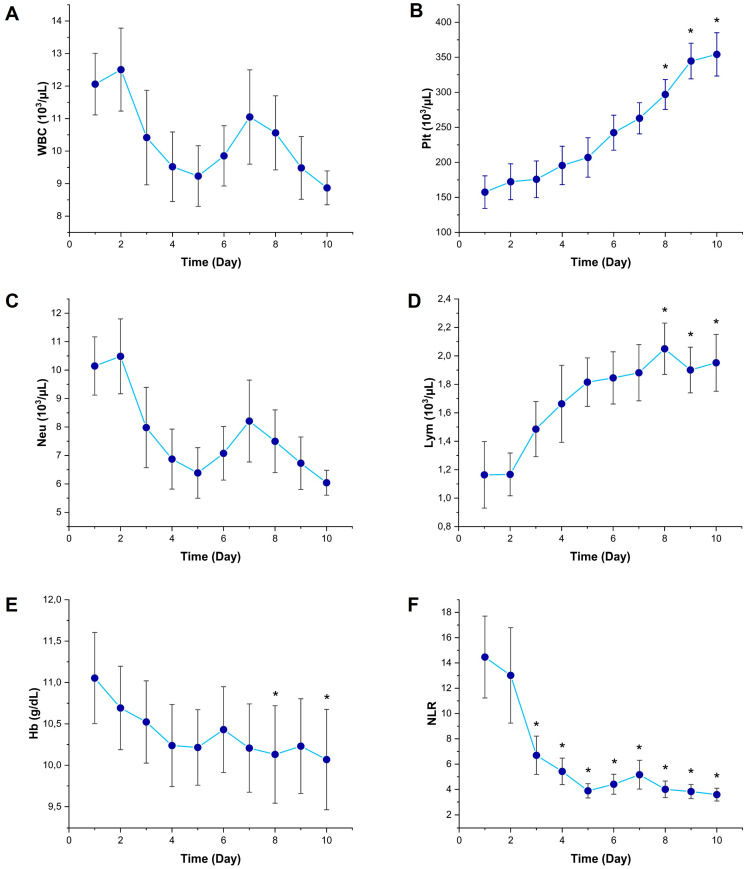
Temporal changes in hematological parameters in sepsis patients. Line graphs showing the daily values of (**A**) WBC (white blood cell count), (**B**) Plt (platelets), (**C**) Neu (neutrophil count), (**D**) Lym (lymphocyte count), (**E**) Hb (hemoglobin count), and (**F**) NLR (neutrophil-to-lymphocyte ratio). Data are presented as mean and SD. * *p* < 0.05 vs. Day 1 by Friedman test followed by Dunn’s multiple comparisons post hoc test.

WBC counts decreased from Day 3 onward, in a manner suggestive of a suppressed leukocyte response during the progression of sepsis; however, no statistically significant difference was found compared to Day 1 (*p* > 0.05) (Figure 2A). Plt counts gradually increased throughout the 10-day follow-up period and reached significantly higher levels on Days 8, 9, and 10 compared to Day 1 (*p* < 0.05); this finding is consistent with a compensatory thrombopoietic response (Figure 2B). Neu counts decreased from Day 3 onward, but no statistically significant difference was observed in comparison to Day 1 (*p* > 0.05) (Figure 2C). Lym counts began to rise starting from Day 3 and were significantly higher on Days 8, 9, and 10 compared to Day 1 (*p* < 0.05); this finding indicates a compensatory recovery within the immune system (Figure 2D). A slight decreasing trend was observed in Hb levels, and this decline reached statistical significance on Days 8 and 10 (*p* < 0.05), which may indicate inflammatory anemia associated with sepsis (Figure 2E). NLR showed a rapid and persistent decline starting from Day 3, and was found to be significantly lower than Day 1 on all subsequent days (*p* < 0.05); this finding highlights the importance of NLR as a dynamic biomarker of systemic inflammation (Figure 2F).

### 3.2. Temporal Changes in Kidney Function Markers

**Figure 3:**
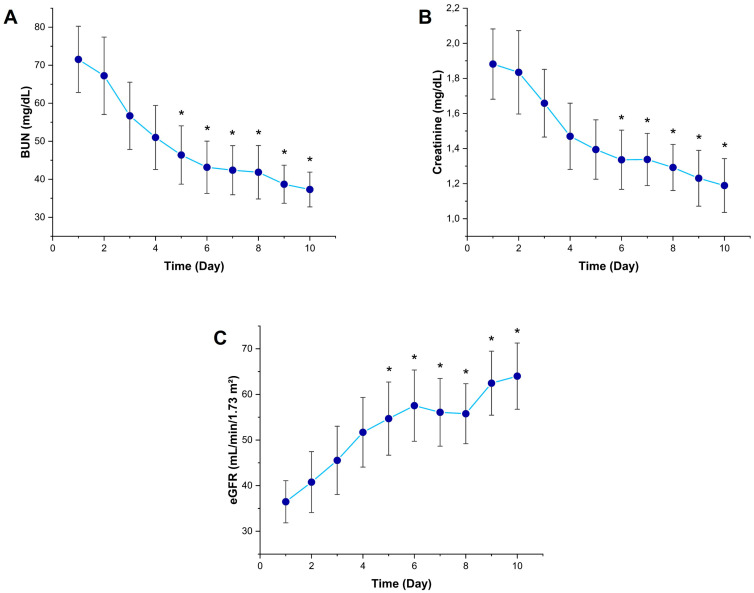
Temporal changes in kidney function biomarkers in sepsis patients. Line graphs showing (**A**) BUN (blood urea nitrogen), (**B**) serum creatinine, and (**C**) Egfr (estimated glomerular filtration rate) levels over 10 days. Data are presented as mean and SD. * *p* < 0.05 vs. Day 1 by Friedman test followed by Dunn’s multiple comparisons post hoc test.

Significant temporal changes were observed in kidney function tests during the 10-day follow-up period. Serum BUN levels began to decrease from Day 2 and were statistically significantly lower than Day 1 from Day 5 onward (*p* < 0.05) (Figure 3A). Similarly, creatinine levels started to show a decreasing trend from Day 2 and were significantly lower than Day 1 on Day 6 and subsequent days (*p* < 0.05) (Figure 3B). eGFR values increased significantly from Day 5 compared to Day 1 (*p* < 0.05), indicating a recovery process in glomerular function following the acute phase of sepsis (Figure 3C).

### 3.3. Correlation of GDF15 with Inflammatory and Renal Biomarkers

**Figure 4:**
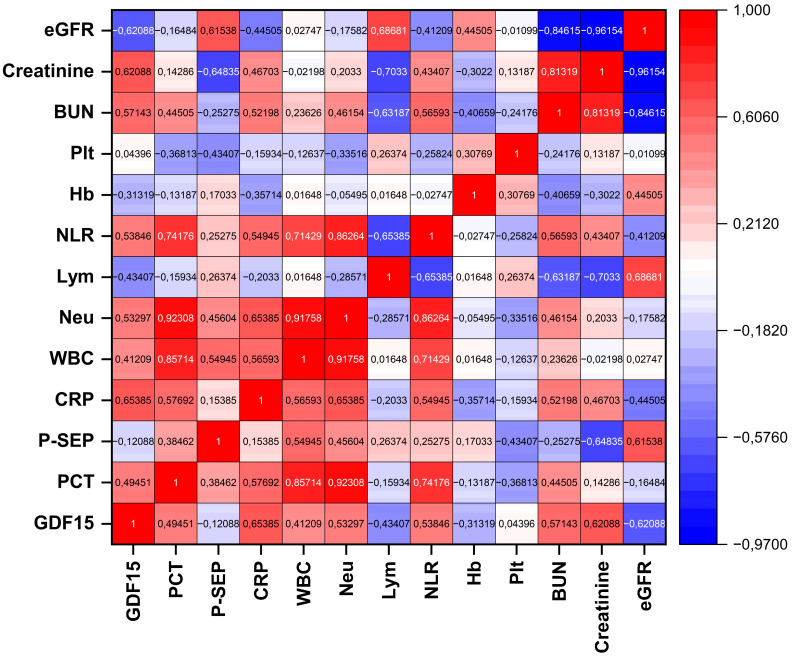
Correlation heat map between GDF15 and clinical biomarkers. Color scale: blue to red = negative to positive.

In the correlation analysis based on the mean values calculated over the 10-day follow-up period, a significant and positive correlation was found between GDF15 and CRP (r = 0.65385, *p* = 0.01535), indicating a moderate association between GDF15 and systemic inflammation. Moderate and statistically significant positive correlations were also observed between GDF15 and BUN (r = 0.57143, *p* = 0.04134) and serum creatinine (r = 0.62088, *p* = 0.02354), demonstrating that GDF15 levels are related to parameters reflecting renal dysfunction. In contrast, a significant negative correlation was found between GDF15 and eGFR (r = −0.62088, *p* = 0.02354), supporting the idea that GDF15 may serve as an indicator of reduced glomerular filtration rate. Although moderate positive correlations were observed between GDF15 and PCT (r = 0.49451) and Neu (r = 0.53297), these associations did not reach statistical significance (*p* = 0.08582 and *p* = 0.06074, respectively). Similarly, the negative correlations between GDF15 and Lym (r = −0.43407, *p* = 0.13834) and Hb (r = −0.31319, *p* = 0.29744) were not statistically significant. Furthermore, no significant relationships were identified between GDF15 and P-SEP (r = −0.12088, *p* = 0.69405), Plt (r = 0.04396, *p* = 0.88662), or WBC (r = 0.41209, *p* = 0.16175). Pairwise relationships are visualized in a correlation heat map, where warmer colors indicate positive and cooler colors indicate negative Spearman correlations; color intensity reflects the magnitude of the coefficient (Figure 4).

## 4. Discussion

At the molecular level, the pathophysiology of sepsis is shaped by excessive activation of the innate immune system. Pathogen-associated molecular patterns (PAMPs) and endogenous structures associated with cellular damage (DAMPs) are primarily recognized by Toll-like receptors, leading to the activation of key transcription factors such as nuclear factor-κB (NF-κB). This activation triggers inflammatory gene expression characterized by excessive cytokine release, including tumor necrosis factor-alpha, interleukin-1 beta, and interleukin-6, ultimately resulting in widespread tissue damage, increased vascular permeability, and multiple organ failure [1,19,20,21].

In this study, the temporal profile of serum GDF15 levels in septic patients was comprehensively evaluated in conjunction with classical inflammatory biomarkers, including CRP, PCT, and P-SEP. Our findings demonstrate that GDF15 levels undergo dynamic changes during the disease. Notably, the significant decrease in GDF15 levels starting from Day 3 and becoming more pronounced after Day 7 suggests a potential compensatory or anti-inflammatory role of this molecule during the resolution phase of sepsis. During the same period, CRP and PCT levels also declined, whereas presepsin levels remained unchanged. These results support the notion that GDF15 is a valuable biomarker reflecting different phases of sepsis with potential utility in clinical monitoring.

Similarly, the study by Li et al. highlighted the diagnostic and prognostic relevance of GDF15 in sepsis patients. GDF15 levels were found to be significantly associated with organ dysfunction, cardiac stress markers, and inflammatory mediators. Moreover, the positive correlations with SOFA score and PCT support the capacity of GDF15 to reflect disease severity and clinical progression. These findings align with our results, which demonstrated dynamic changes in GDF15 levels and their correlation with systemic inflammation. Furthermore, in vitro experiments have demonstrated that GDF15 exhibits anti-inflammatory effects and regulates macrophage polarization, underscoring its pathophysiological significance [16].

Similarly, Buendgens et al. reported that GDF15 levels were significantly elevated in critically ill patients, both with and without sepsis, with more pronounced elevations observed in septic patients. GDF15 was shown to reflect early organ dysfunction and was associated with clinical severity scores. Additionally, elevated GDF15 levels were identified as an independent risk factor for both short- and long-term mortality, further reinforcing the clinical value of GDF15 in line with our findings [15].

Several recent clinical studies have further highlighted the prognostic value of GDF15 in patients with sepsis. Ji et al. demonstrated that elevated serum GDF15 levels were significantly associated with increased 28-day mortality in ICU patients with sepsis, identifying GDF15 as an independent prognostic marker with high predictive accuracy [22].

The study by Lim et al., which explored conditions outside of sepsis, demonstrated a significant relationship between elevated GDF15 levels and disease severity and mortality in patients receiving continuous renal replacement therapy due to acute kidney injury. This study suggests that GDF15 is not only a marker of inflammation but also a comprehensive indicator of systemic pathophysiological processes. Its superior prognostic performance compared to APACHE II and SOFA scores suggests that it may contribute meaningfully to risk assessment tools in clinical practice [23].

Alipanah-Lechner et al. focused on the metabolic relevance of GDF15, reporting that hyperinflammatory phenotypes in ARDS and sepsis patients were characterized by high GDF15 levels, which were associated with mitochondrial stress and impaired lipid metabolism. This study suggests that GDF15 may represent a unique pathophysiological pathway independent of inflammation and disease severity. Accordingly, it offers a valuable perspective on the underlying metabolic and cellular stress mechanisms that may explain the dynamic changes in GDF15 observed in our study [24].

Additionally, supporting mechanistic insights from animal studies, such as those by Chen et al. and Zhang et al., further suggest that GDF15 may exert protective effects by modulating macrophage polarization and influencing neuroinflammatory responses, highlighting its complex and multifaceted biological role in the pathophysiology of sepsis [12,25]. For instance, Li et al. demonstrated that exogenous GDF15 administration significantly reduced liver injury and systemic inflammation in LPS-induced endotoxemia by inhibiting the TAK1–NF-κB signaling pathway in Kupffer cells, highlighting GDF15’s anti-inflammatory potential [26]. Similarly, Lu et al. demonstrated that GDF15 alleviated sepsis-induced lung injury by inhibiting glycolysis through AMPK-mediated mechanisms in alveolar macrophages, thereby suppressing the NF-κB and MAPK inflammatory pathways, and underscoring its role in metabolic modulation during inflammatory stress [27]. Li et al. also reported the protective cardiac effects of GDF15 in septic cardiomyopathy models, demonstrating that GDF15 prevents cardiomyocyte ferroptosis via a SOCS1/GPX4-dependent mechanism, which indicates its potential therapeutic value for organ protection during sepsis [28]. Additionally, Abulizi et al. provided evidence that GDF15 deficiency exacerbated inflammatory responses and worsened kidney and cardiac injury in septic mice, affirming the essential role of GDF15 in limiting excessive immune activation and tissue damage during systemic inflammation [29].

Lastly, the absence of a significant correlation between GDF15 and presepsin in our study, as well as the lack of such a relationship reported in the literature, is noteworthy. While presepsin rises rapidly during early sepsis and then stabilizes, GDF15 demonstrates a delayed decrease, suggesting that these two biomarkers may have distinct temporal kinetics and reflect different pathophysiological aspects of sepsis. This divergence represents an important research question for future comparative studies [30].

Our data do not permit claims about the “superiority” of any single biomarker for sepsis diagnosis or monitoring. The cohort size, the absence of control groups, and our exploratory design preclude valid estimates of sensitivity/specificity; accordingly, we avoided ranking markers to prevent overinterpretation. In our 10-day trajectories, GDF15 exhibited a later and more gradual decline than CRP and PCT. At the same time, presepsin showed no significant temporal change—consistent with GDF15 indexing sustained host stress/organ involvement rather than the earlier inflammatory dynamics captured by CRP/PCT. These findings support a multi-biomarker strategy in which GDF15 is interpreted together with established markers to reflect clinical course and organ dysfunction better. Definitive comparisons will require larger, multicenter cohorts with appropriate control groups (e.g., non-infectious SIRS and healthy comparators) and multivariable longitudinal analyses (e.g., mixed-effects regression) alongside diagnostic accuracy studies (ROC/AUC with standardized cut-offs).

By way of illustration, in a patient with urosepsis and sepsis-associated acute kidney injury, a consistent fall in GDF15 from approximately Day 3 onward—paralleling declines in creatinine/BUN and an increase in eGFR—would support effective source control and renal recovery and could inform step-down decisions. Conversely, discordance between biomarkers (e.g., falling CRP/PCT but persistently elevated or rising GDF15 with stagnant eGFR) may flag ongoing organ stress and prompt re-evaluation of volume status, nephrotoxic exposures, or occult complications. This example is hypothesis-generating and requires validation in larger cohorts.

Although this study presents important findings, it also has several limitations. First, the relatively small sample size (*n* = 13) restricts the generalizability of the findings. In addition, because the number and distribution of pathogens were small and heterogeneous, the study was underpowered to assess organism-specific differences in biomarker trajectories; exploratory inspection did not reveal consistent pathogen-dependent patterns, and we therefore did not perform formal subgroup analyses. The inclusion criterion based on culture positivity limits the study population to sepsis cases with clearly identified sources of infection, potentially reducing applicability to broader septic populations. Moreover, the presence of multiple comorbidities (e.g., hypertension, diabetes, renal failure) in all patients may have influenced GDF15 levels. Nevertheless, this reflects a real-world sepsis profile. Only serum-based analyses were performed; no advanced molecular analyses were conducted to assess tissue-level expression or determine the cellular source of GDF15. Finally, the study was single-centered and requires validation through larger, multicenter prospective studies.

## 5. Conclusions

This study provides preliminary data revealing the temporal pattern of GDF15 levels and its relationship with classical inflammatory biomarkers in patients with sepsis. The significant decline in GDF15 levels over the 10-day follow-up and its meaningful correlations with CRP and renal dysfunction indicators such as BUN, creatinine, and eGFR suggest that this biomarker may reflect not only the inflammatory response but also organ damage in sepsis. Our findings indicate that GDF15 may serve as a valuable biomarker for both monitoring the resolution phase of inflammation and assessing renal dysfunction in the clinical management of sepsis.

## Data Availability

The original contributions presented in this study are included in the article; further inquiries can be directed to the corresponding author.

## Supplementary Materials

The following supporting information can be downloaded at https://www.mdpi.com/article/10.3390/diagnostics15172224/s1. Detailed demographic, clinical, and biochemical data of the patients included in this study are presented in the Supplementary Materials section (see Supplementary Figures S1–S13).
