# Supplementary material for: Is GDF15 a Feasible Biomarker in Sepsis?
Source: Diagnostics (Basel). 2025 Sep 2;15(17):2224. doi: 10.3390/diagnostics15172224 (PMC12428078; doi:10.3390/diagnostics15172224)
Supplement: Supplementary file 1 [file diagnostics-15-02224-s001.zip › diagnostics-3770446-SI.pdf]

Patient ID: 1
Gender: Male
Age: 86
BMI: 28.1
Blood Culture: (-)
Urine Culture: (+)
DM (-)
HT (-)
CAD (-)
CKD (+)
Sofa Score: 3

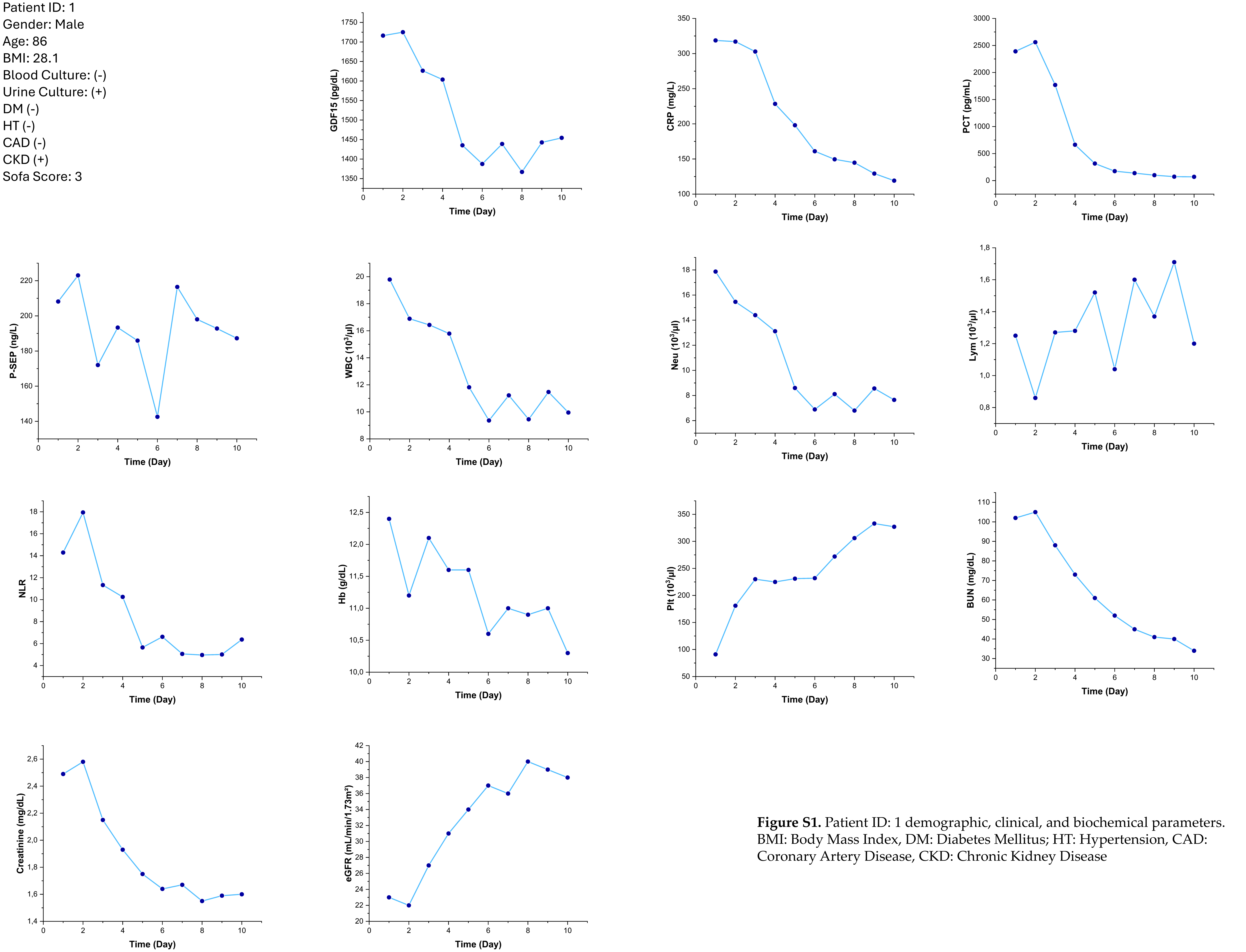

Figure S1. Patient ID: 1 demographic, clinical, and biochemical parameters. BMI: Body Mass Index, DM: Diabetes Mellitus; HT: Hypertension, CAD: Coronary Artery Disease, CKD: Chronic Kidney Disease

Patient ID: 2
Gender: Male
Age: 80
BMI: 25.1
Blood Culture: (-)
Urine Culture: (+)
DM (-)
HT (+)
CAD (-)
CKD (+)
Sofa Score: 3

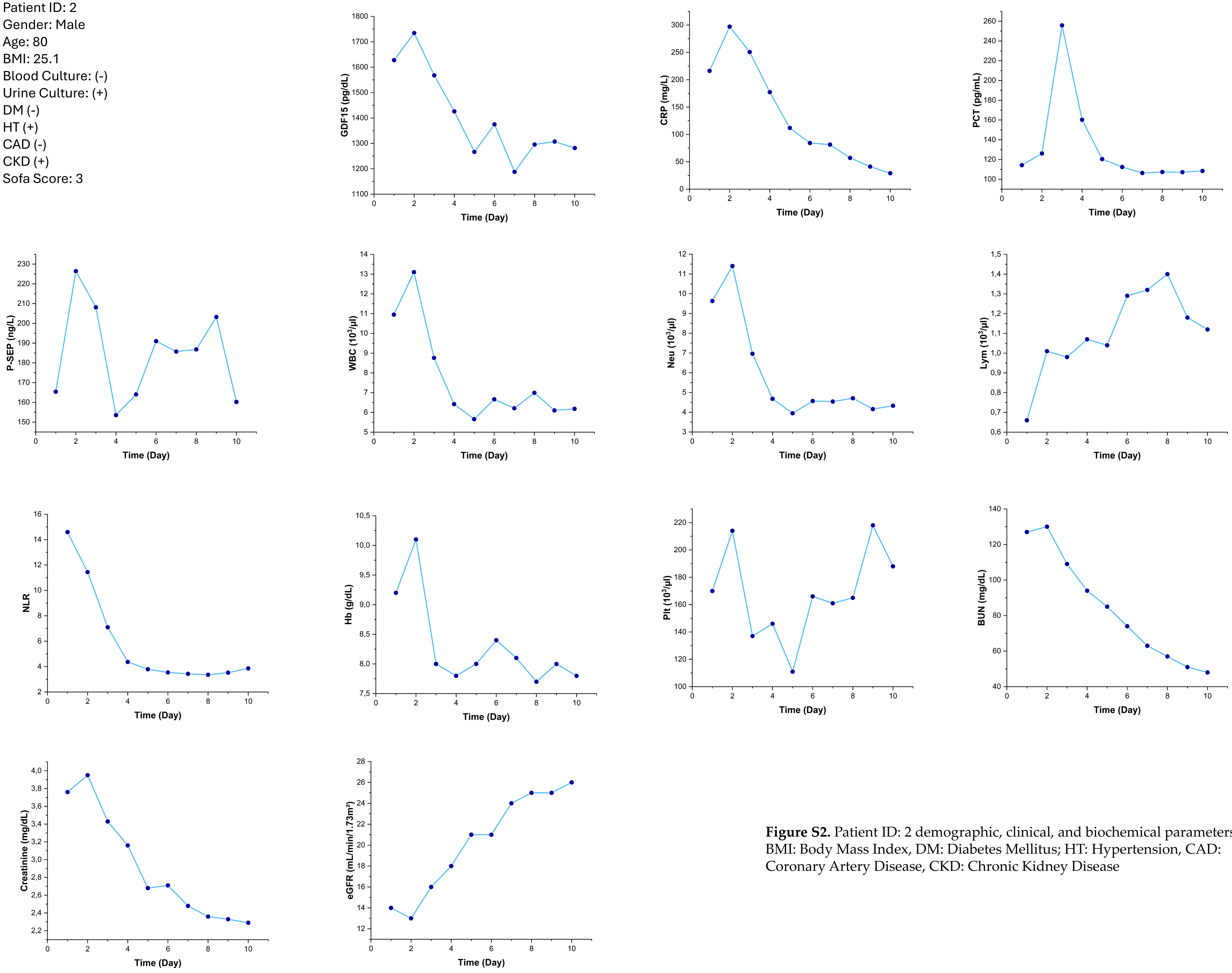

Figure S2. Patient ID: 2 demographic, clinical, and biochemical parameters. BMI: Body Mass Index, DM: Diabetes Mellitus; HT: Hypertension, CAD: Coronary Artery Disease, CKD: Chronic Kidney Disease

Patient ID: 3
Gender: Male
Age: 73
BMI: 31.8
Blood Culture: (-)
Urine Culture: (+)
DM (-)
HT (+)
CAD (+)
CKD (+)
Sofa Score: 2

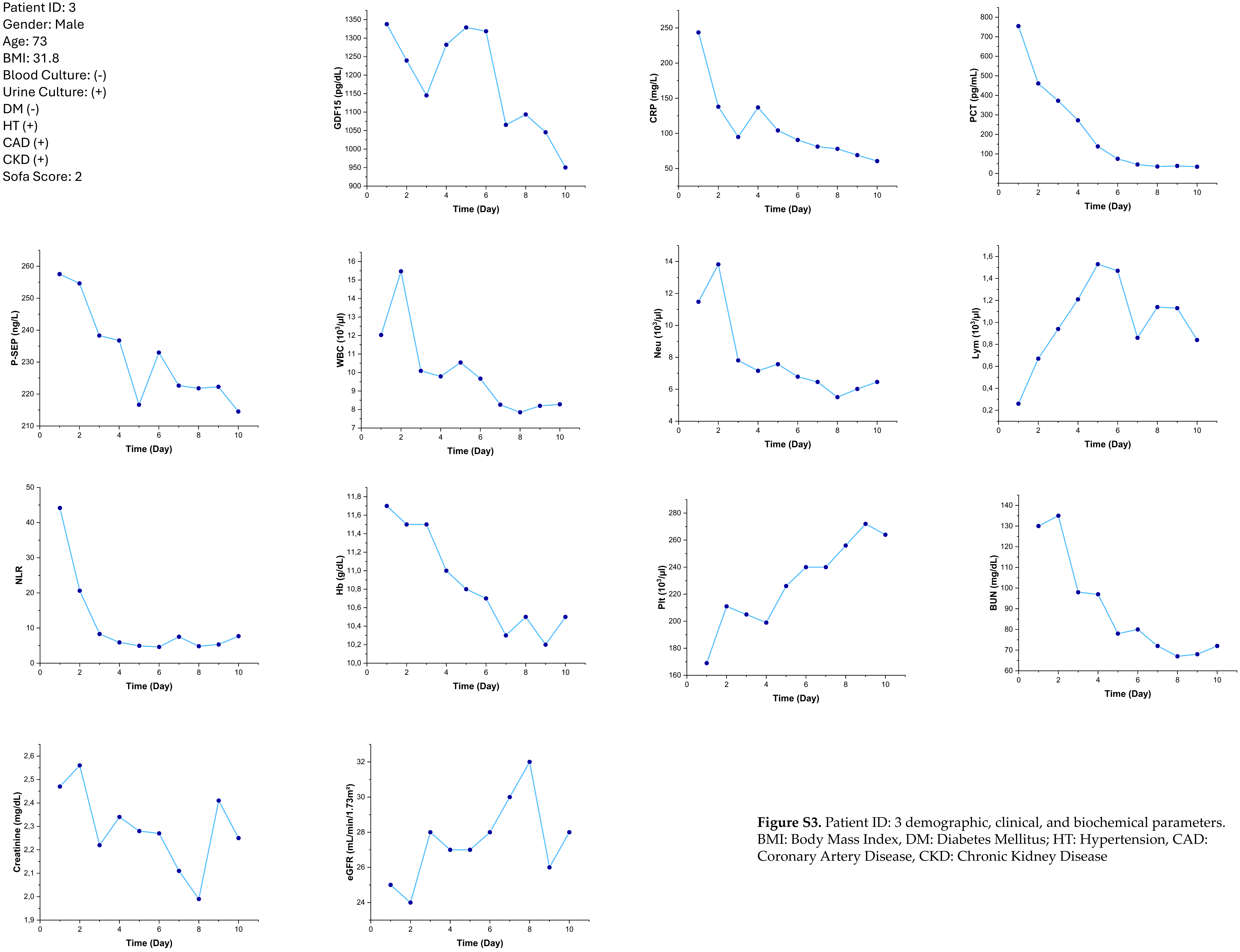

Figure S3. Patient ID: 3 demographic, clinical, and biochemical parameters. BMI: Body Mass Index, DM: Diabetes Mellitus; HT: Hypertension, CAD: Coronary Artery Disease, CKD: Chronic Kidney Disease

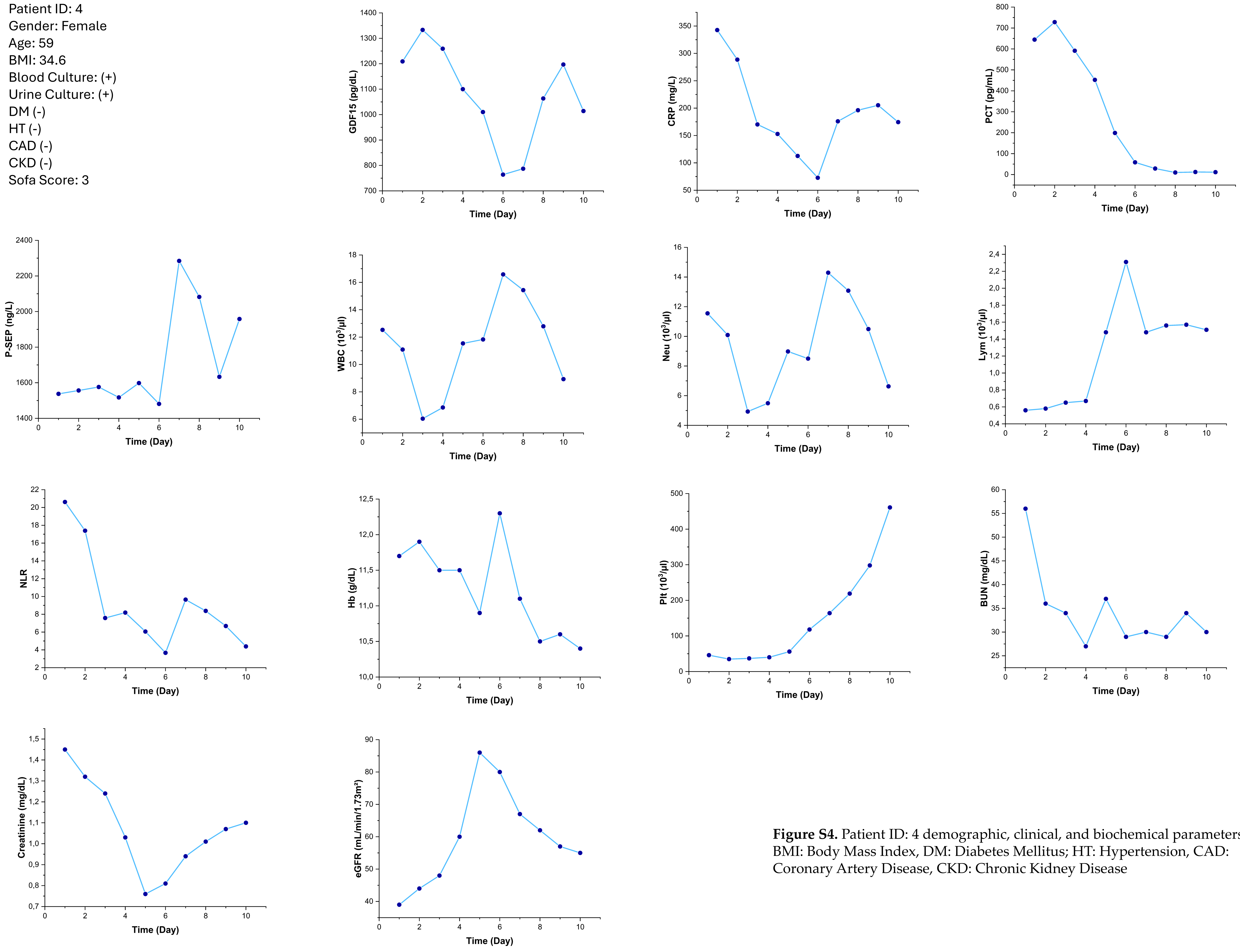

Figure S4. Patient ID: 4 demographic, clinical, and biochemical parameters. BMI: Body Mass Index, DM: Diabetes Mellitus; HT: Hypertension, CAD: Coronary Artery Disease, CKD: Chronic Kidney Disease

Patient ID: 5
Gender: Female
Age: 58
BMI: 29.1
Blood Culture: (-)
Urine Culture: (+)
DM (-)
HT (+)
CAD (-)
CKD (-)
Sofa Score: 2

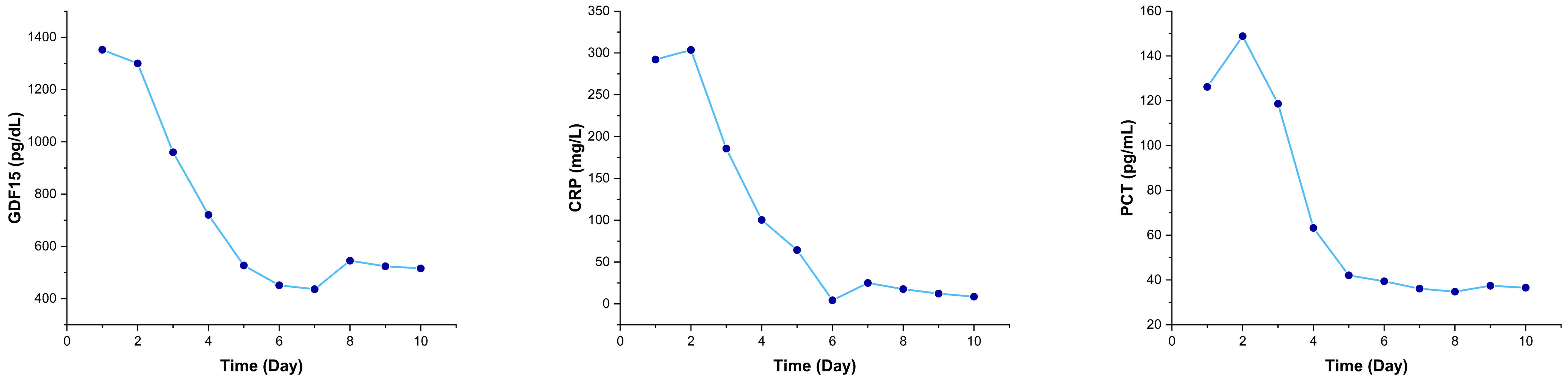

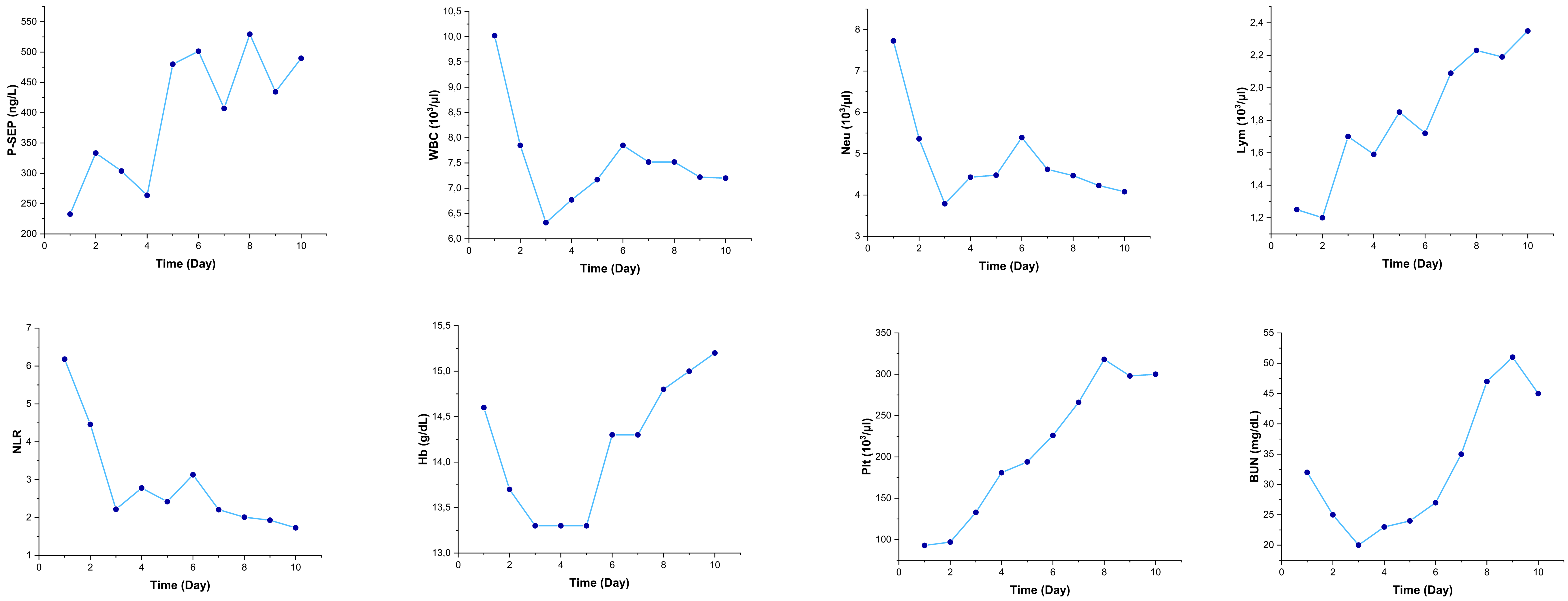

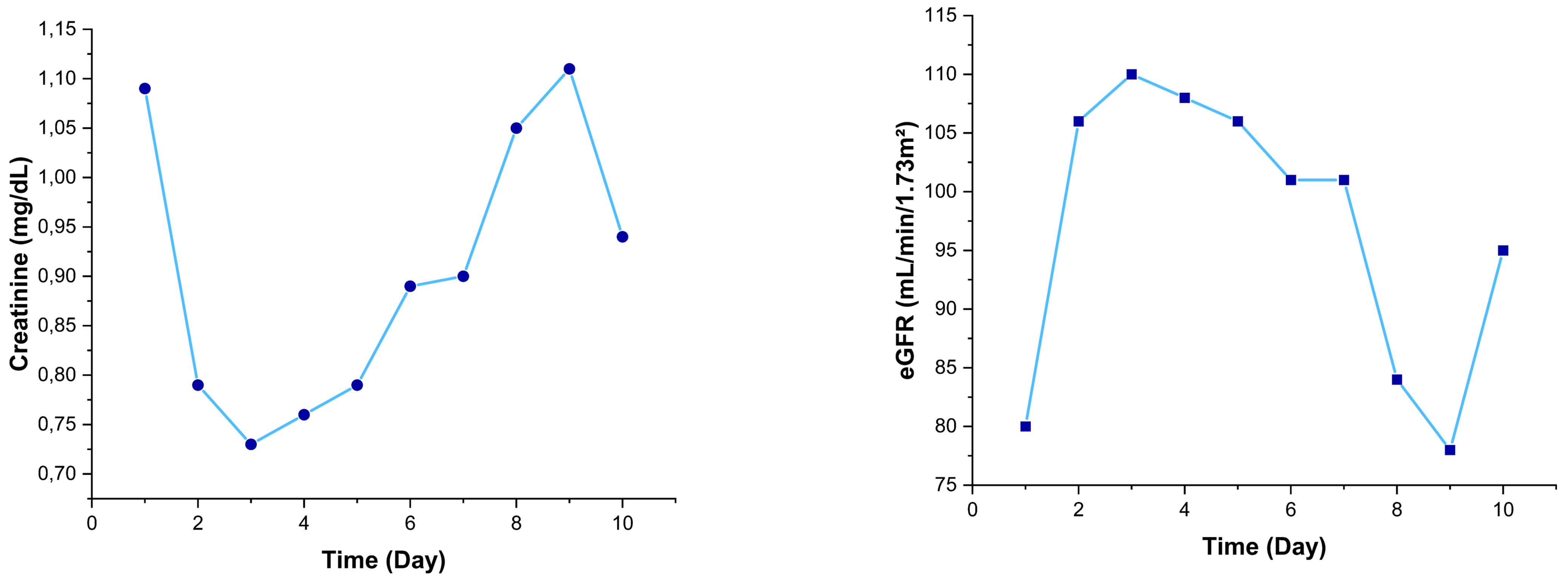

Figure S5. Patient ID: 5 demographic, clinical, and biochemical parameters. BMI: Body Mass Index, DM: Diabetes Mellitus; HT: Hypertension, CAD: Coronary Artery Disease, CKD: Chronic Kidney Disease

Patient ID: 6
Gender: Male
Age: 49
BMI: 26.1
Blood Culture: (-)
Urine Culture: (+)
DM (-)
HT (-)
CAD (-)
CKD (-)
Sofa Score: 2

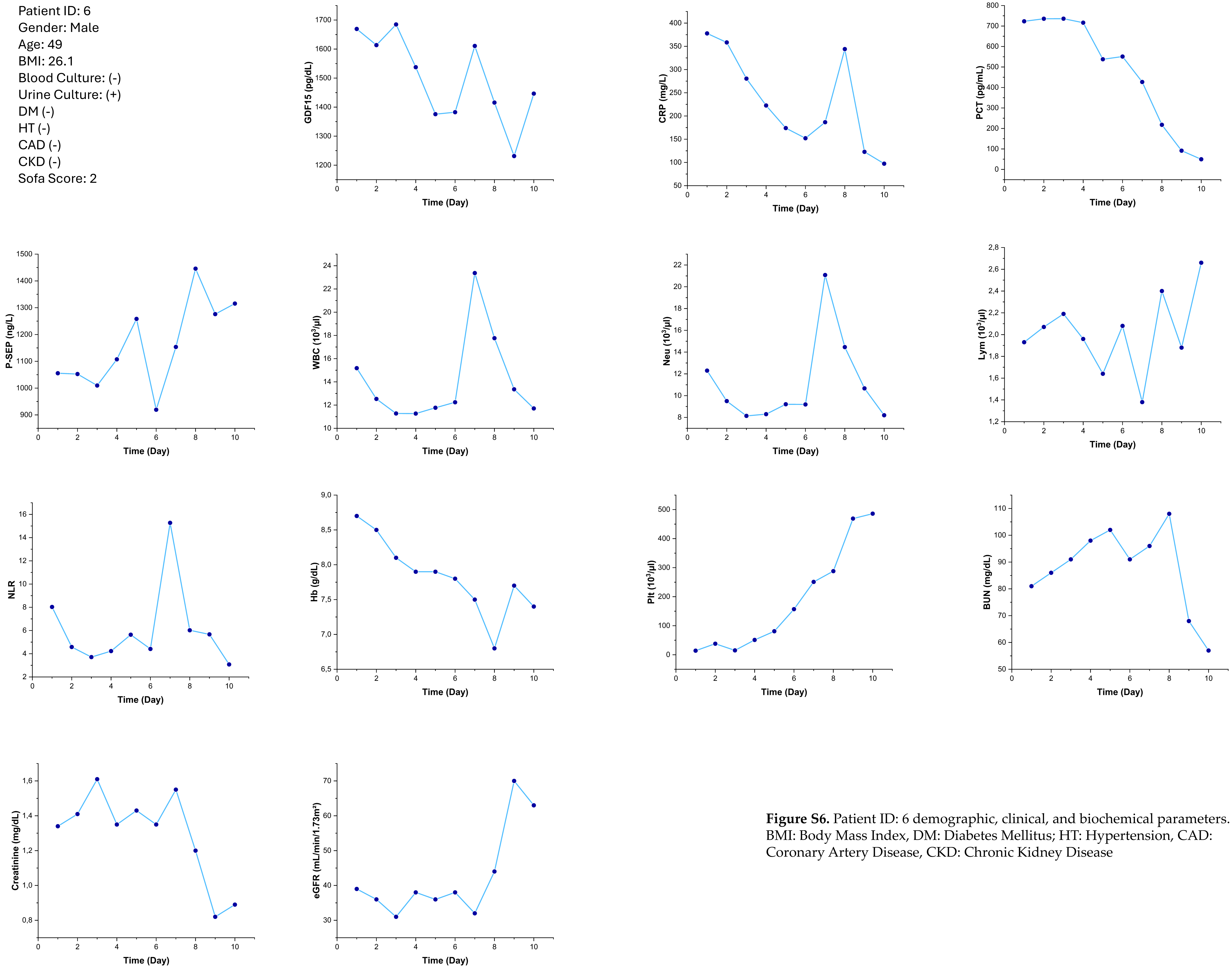

Figure S6. Patient ID: 6 demographic, clinical, and biochemical parameters. BMI: Body Mass Index, DM: Diabetes Mellitus; HT: Hypertension, CAD: Coronary Artery Disease, CKD: Chronic Kidney Disease

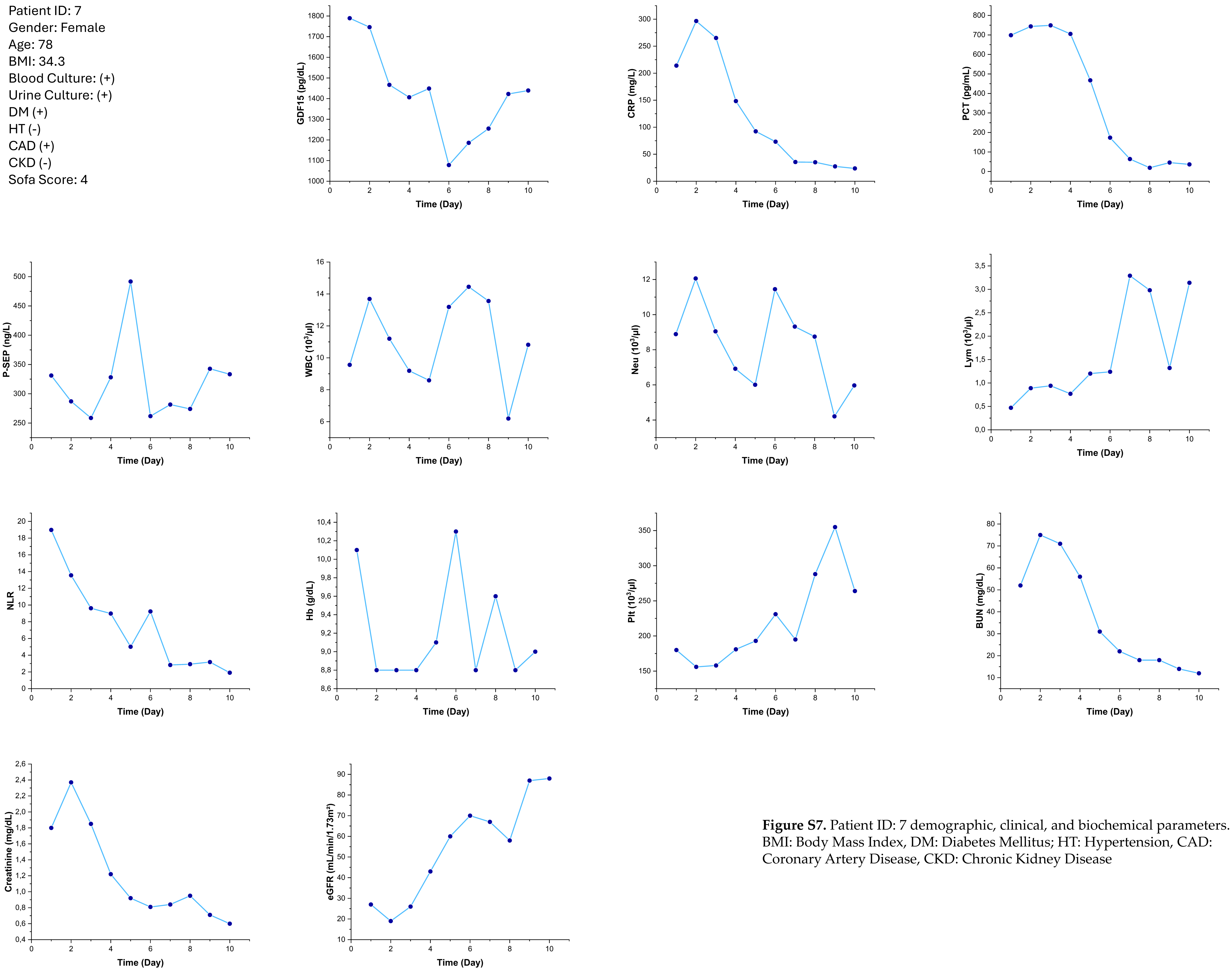

Figure S7. Patient ID: 7 demographic, clinical, and biochemical parameters. BMI: Body Mass Index, DM: Diabetes Mellitus; HT: Hypertension, CAD: Coronary Artery Disease, CKD: Chronic Kidney Disease

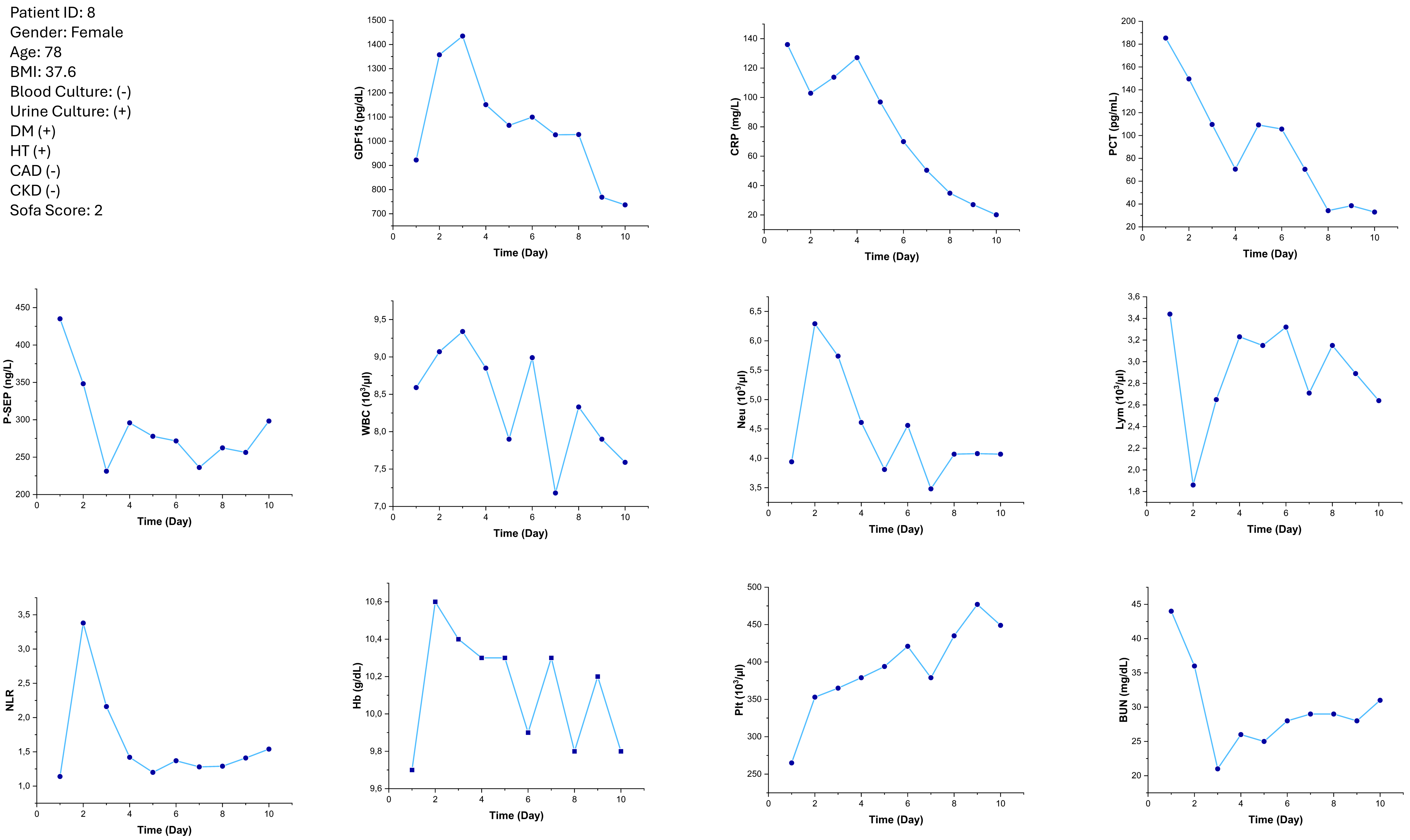

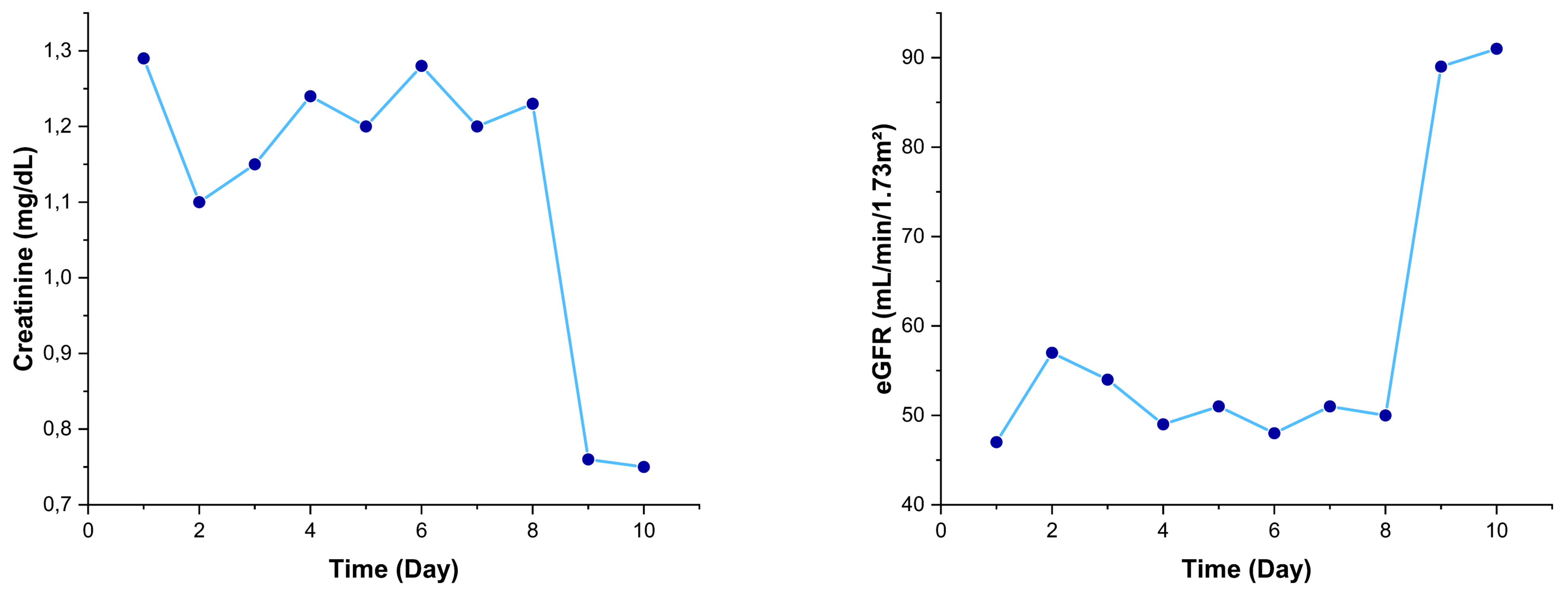

Figure S8. Patient ID: 8 demographic, clinical, and biochemical parameters. BMI: Body Mass Index, DM: Diabetes Mellitus; HT: Hypertension, CAD: Coronary Artery Disease, CKD: Chronic Kidney Disease

Patient ID: 9
Gender: Female
Age: 56
BMI: 26.7
Blood Culture: (-)
Urine Culture: (+)
DM (+)
HT (+)
CAD (-)
CKD (-)
Sofa Score: 2

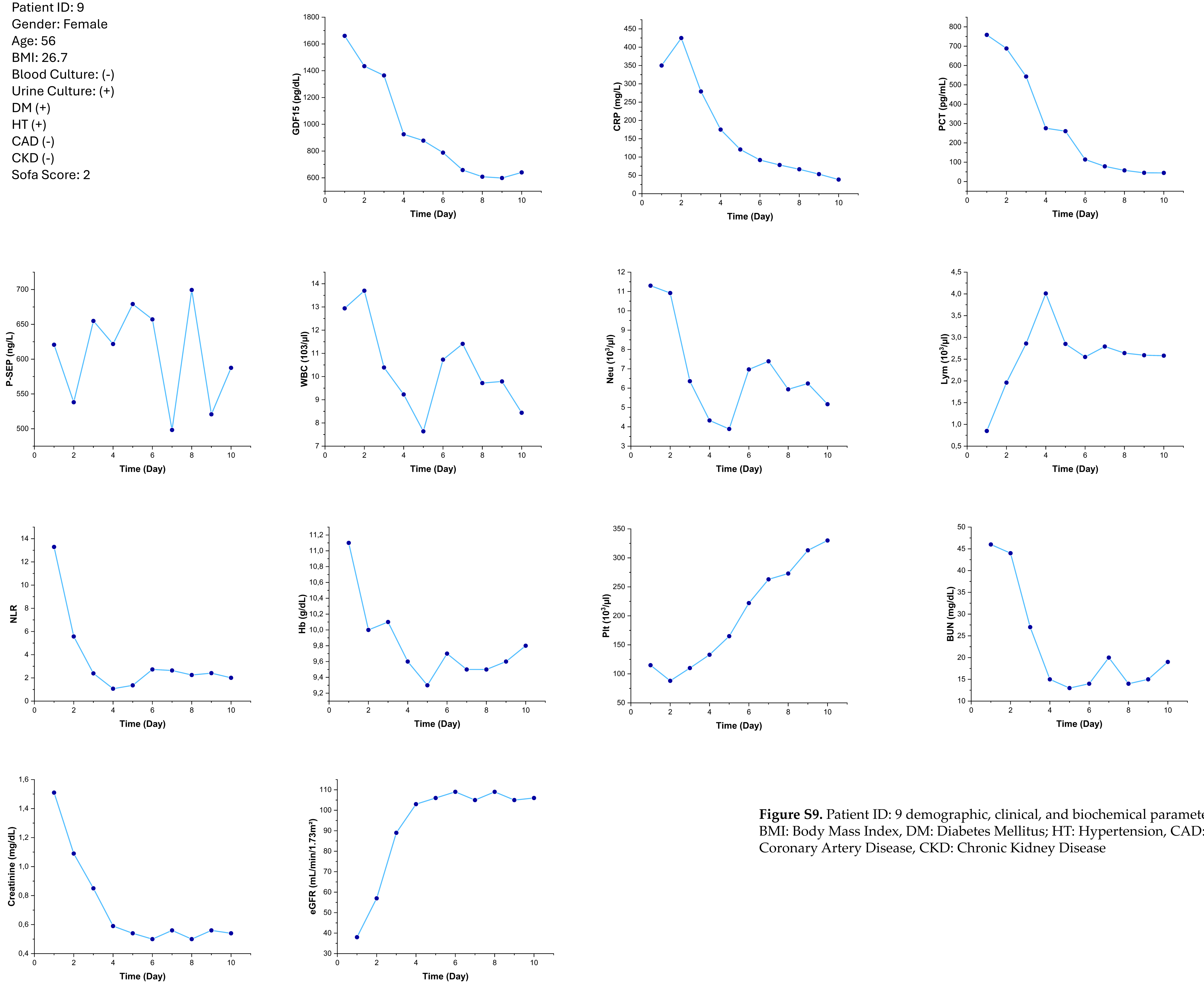

Figure S9. Patient ID: 9 demographic, clinical, and biochemical parameters. BMI: Body Mass Index, DM: Diabetes Mellitus; HT: Hypertension, CAD: Coronary Artery Disease, CKD: Chronic Kidney Disease

Patient ID: 10
Gender: Female
Age: 55
BMI: 29.8
Blood Culture: (-)
Urine Culture: (+)
DM (+)
HT (+)
CAD (-)
CKD (-)
Sofa Score: 2

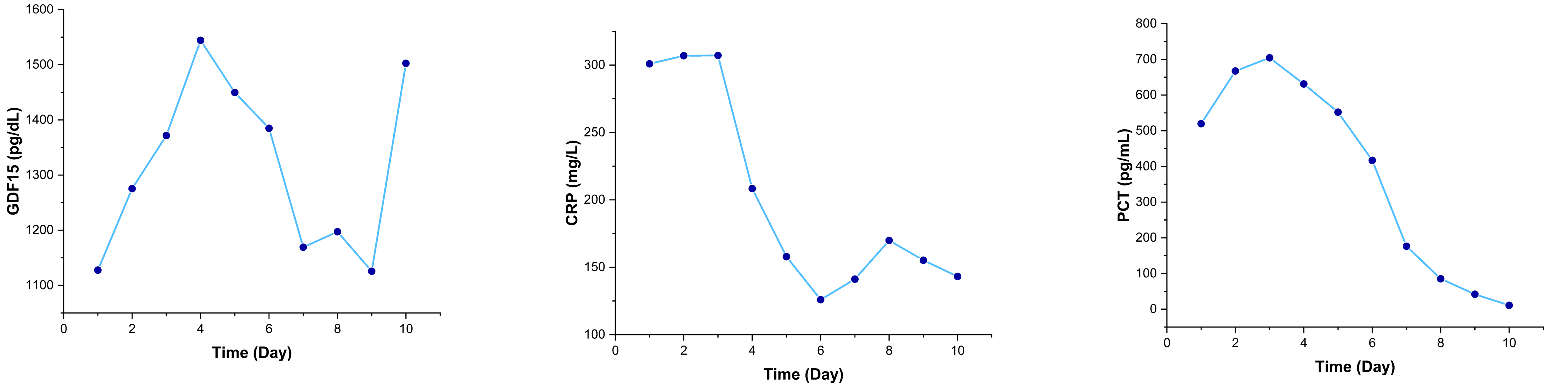

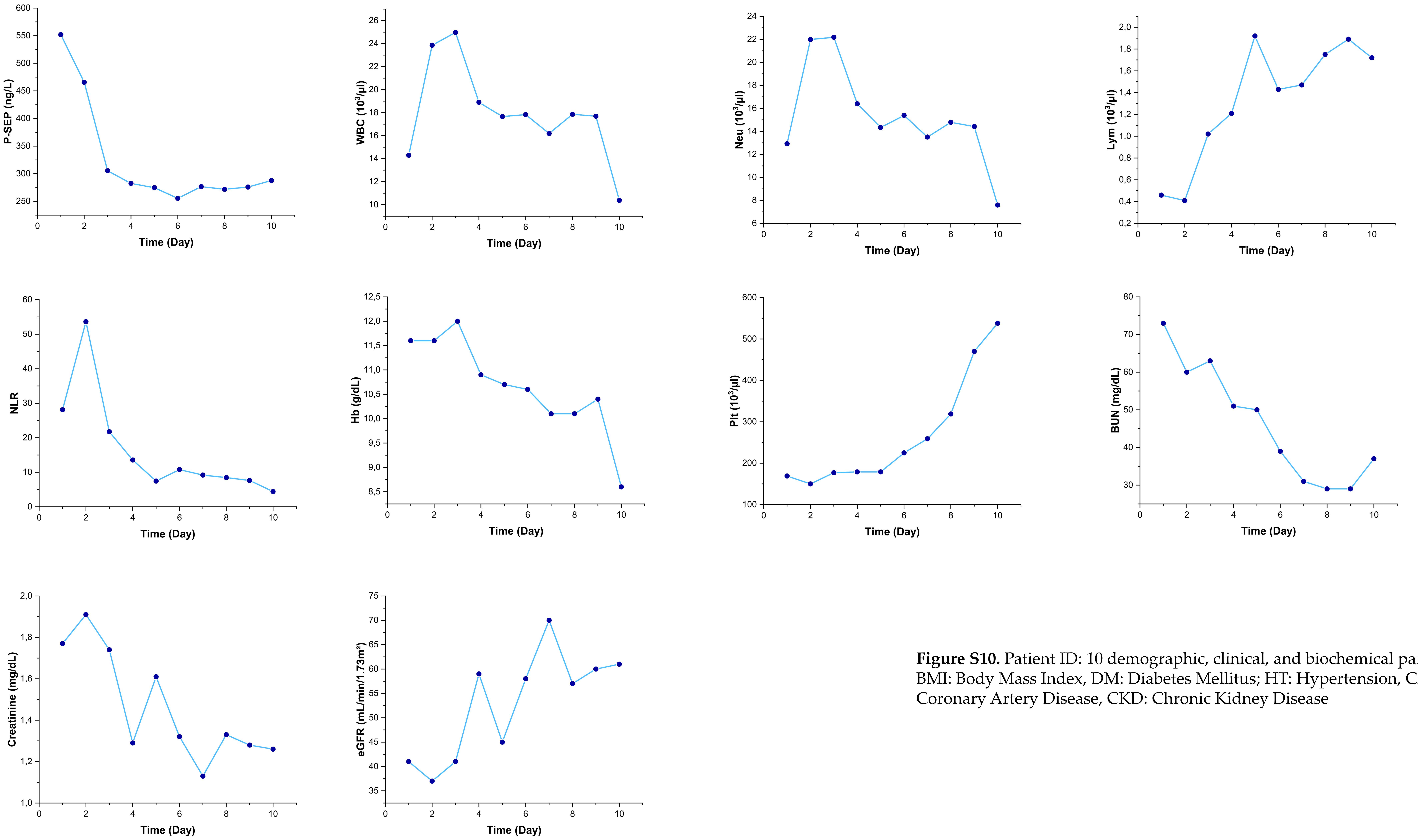

Figure S10. Patient ID: 10 demographic, clinical, and biochemical parameters. BMI: Body Mass Index, DM: Diabetes Mellitus; HT: Hypertension, CAD: Coronary Artery Disease, CKD: Chronic Kidney Disease

Patient ID: 11
Gender: Male
Age: 59
BMI: 29.1
Blood Culture: (-)
Urine Culture: (+)
DM (+)
HT (+)
CAD (-)
CKD (-)
Sofa Score: 3

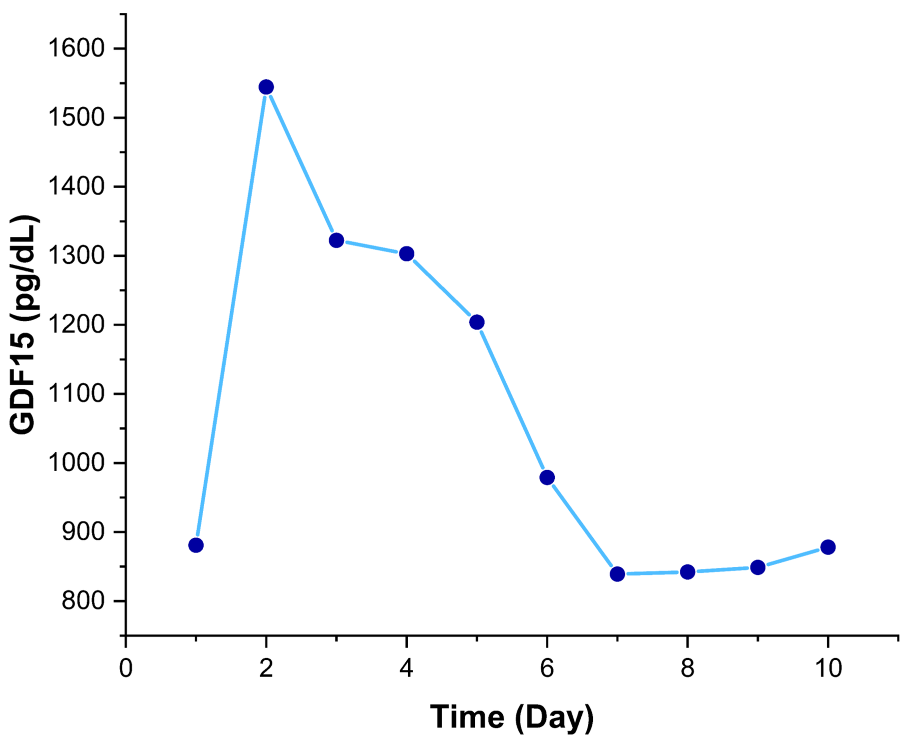

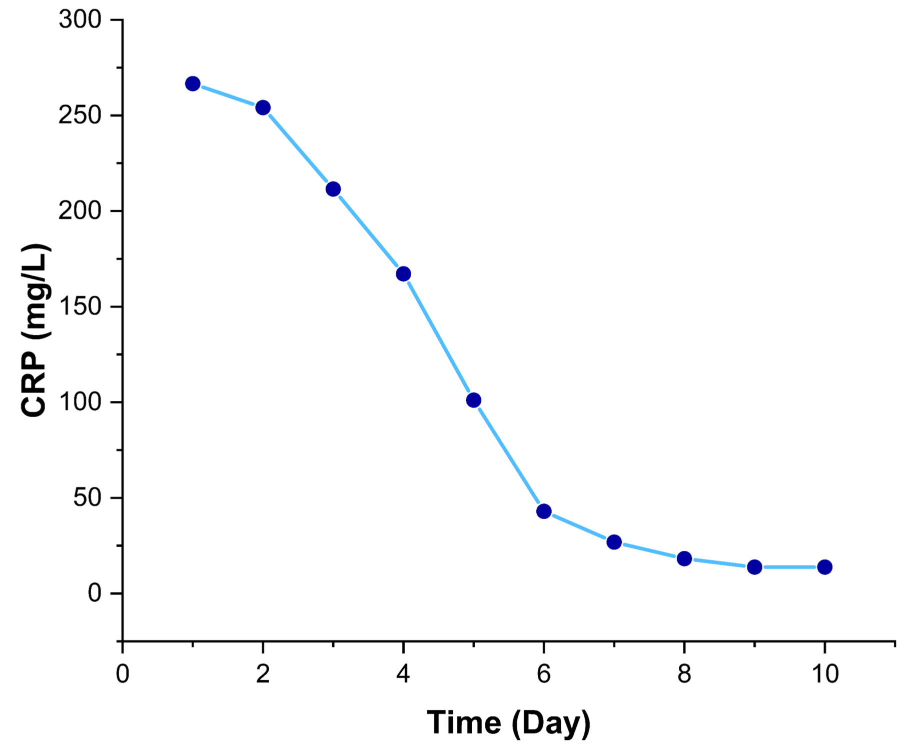

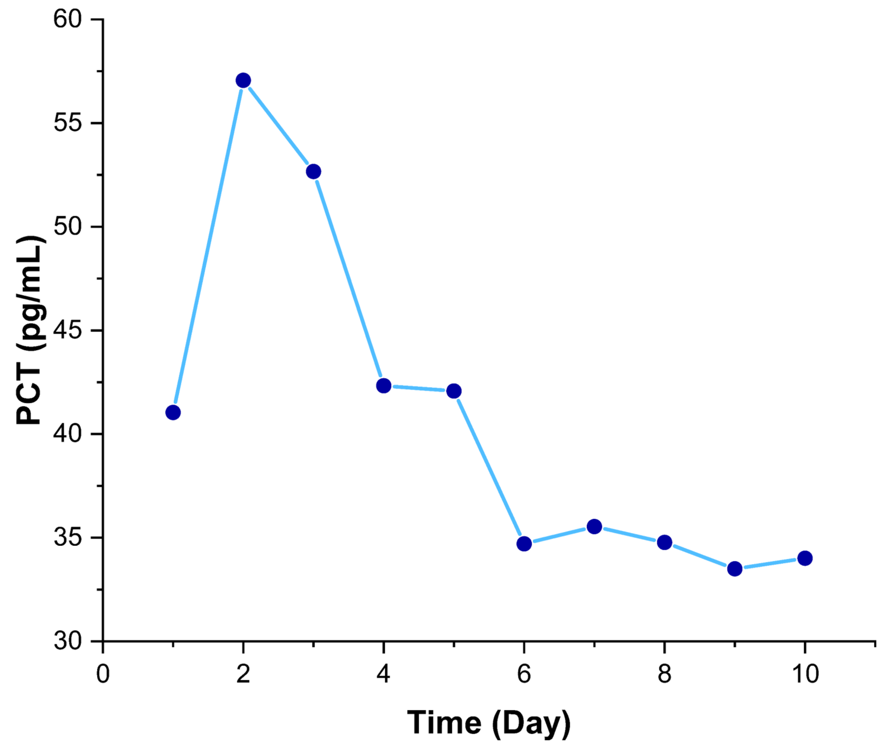

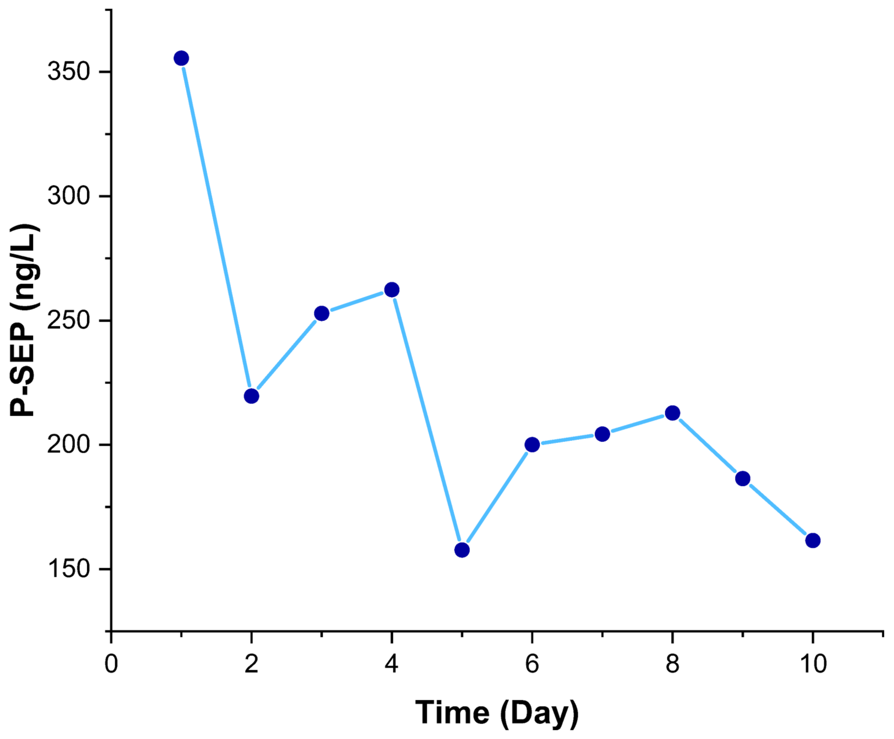

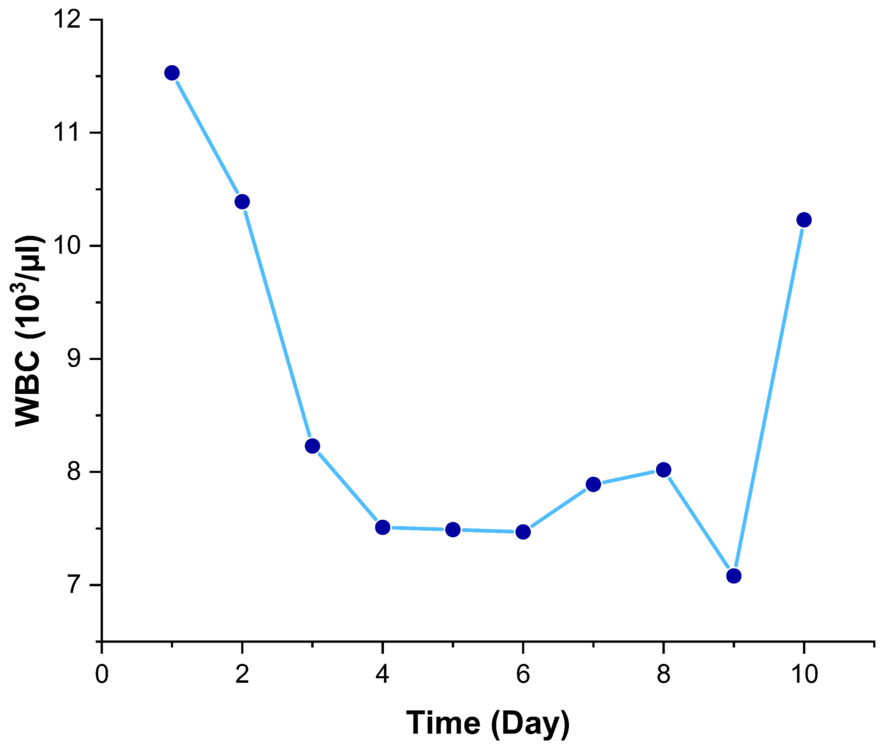

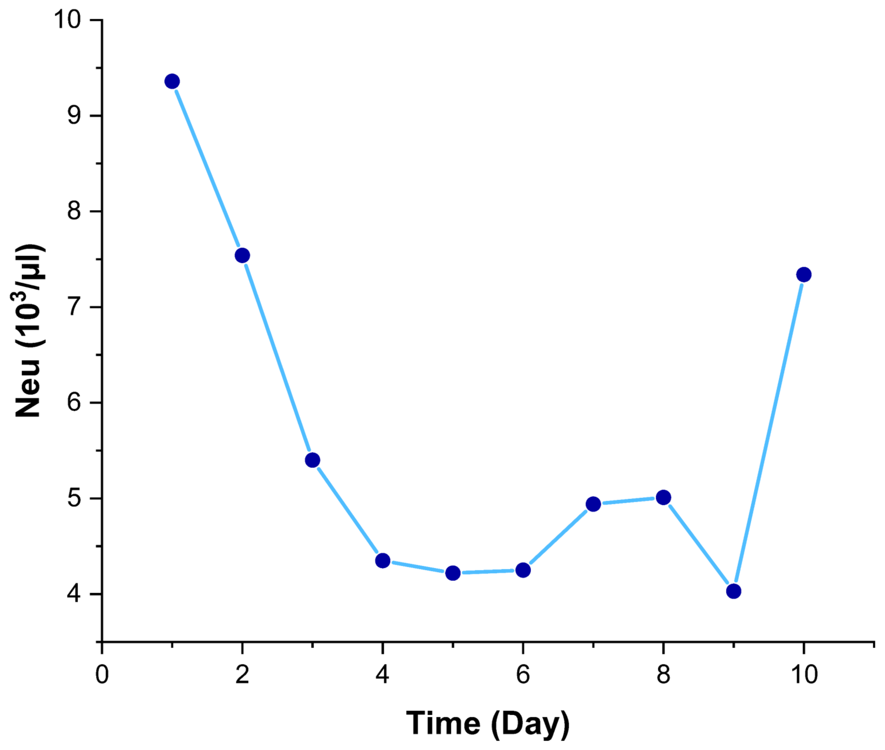

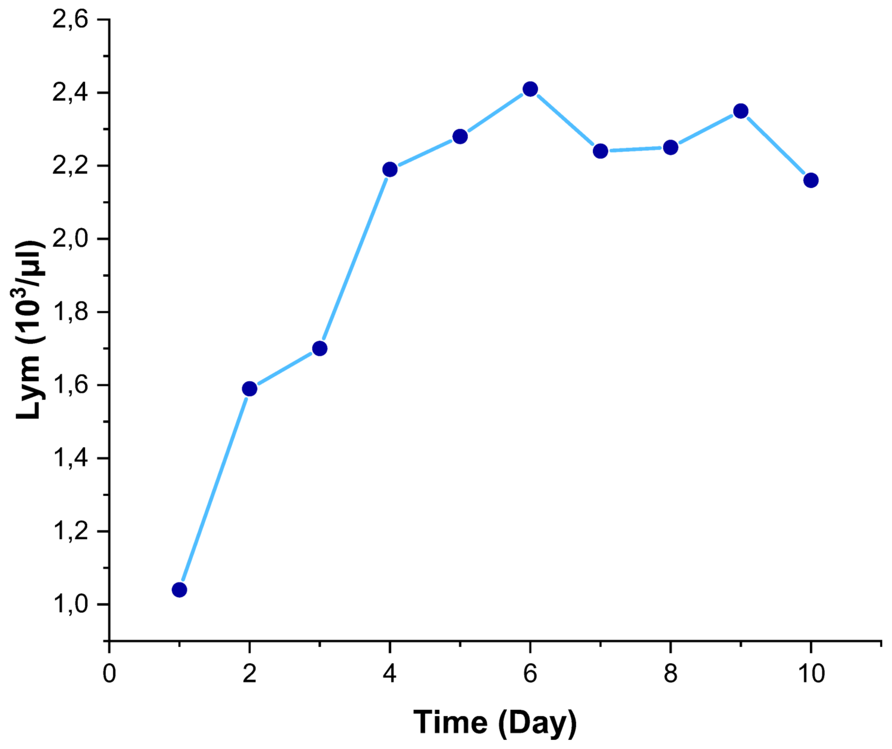

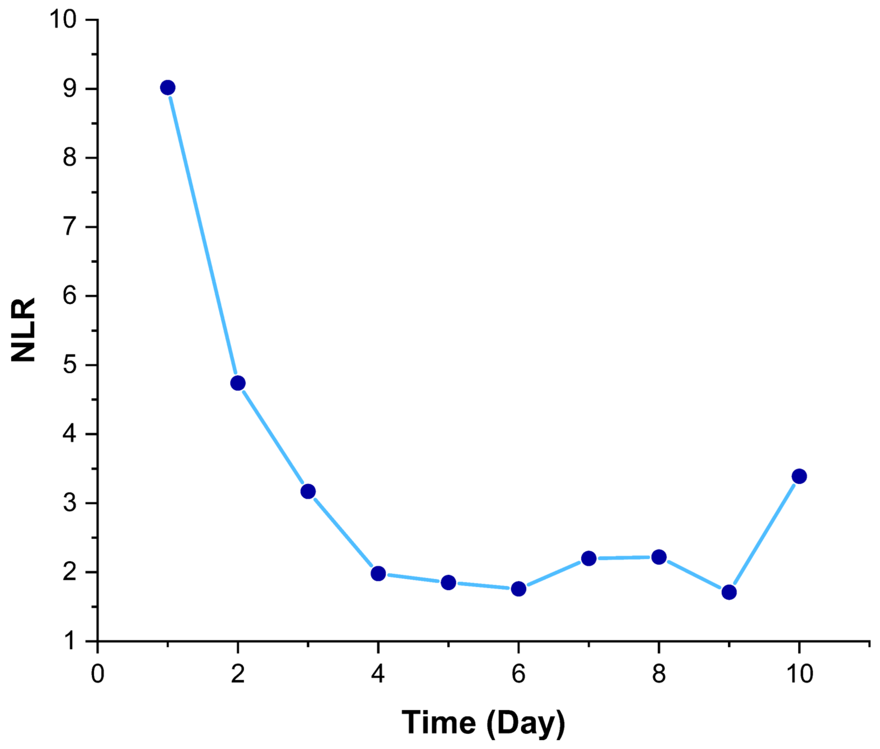

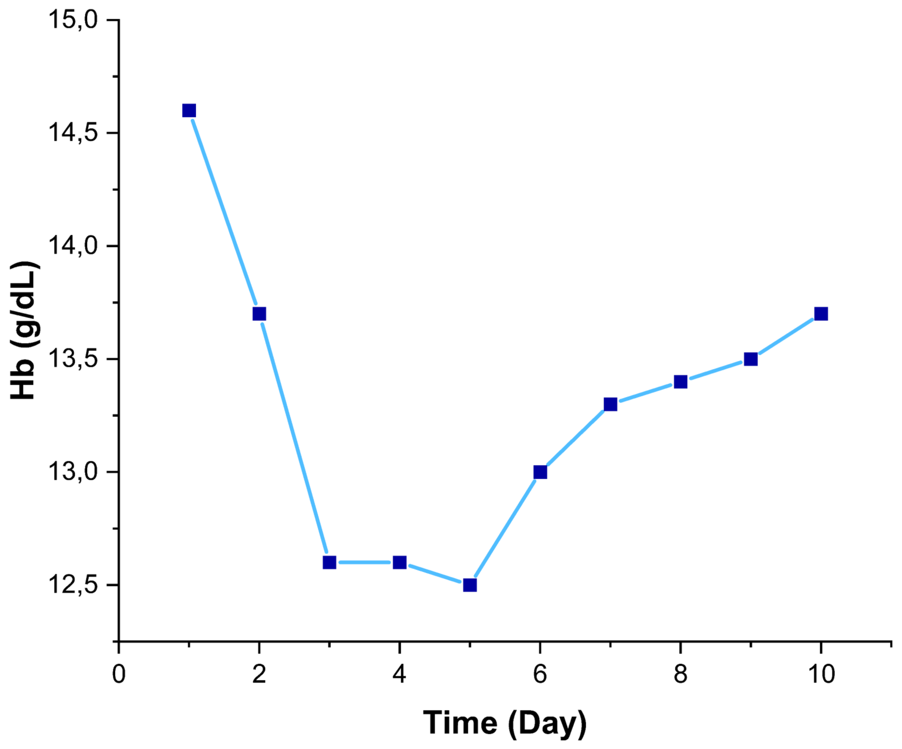

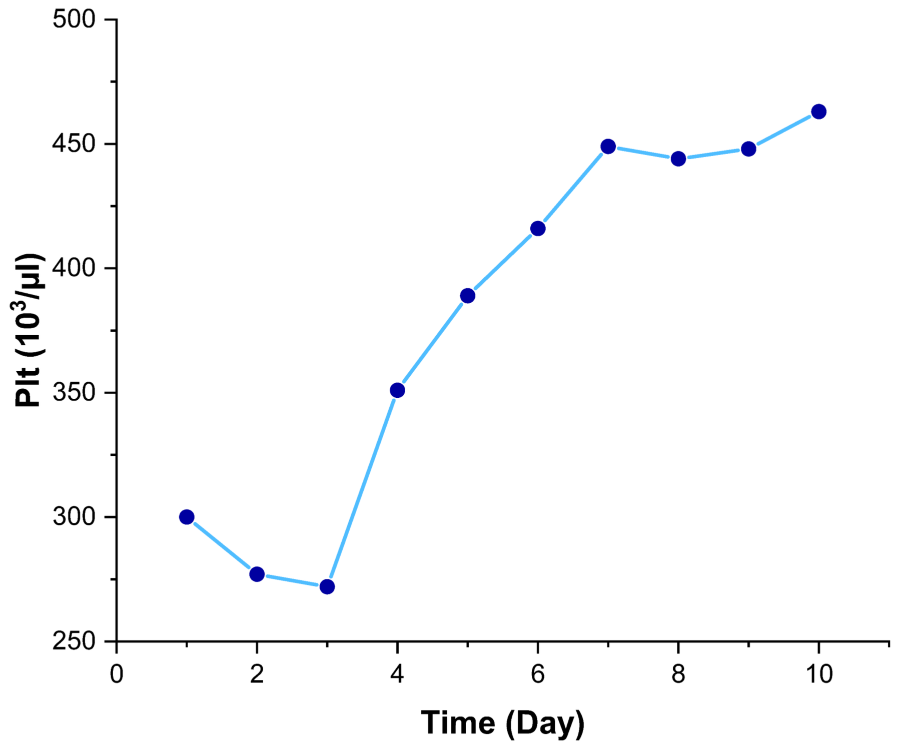

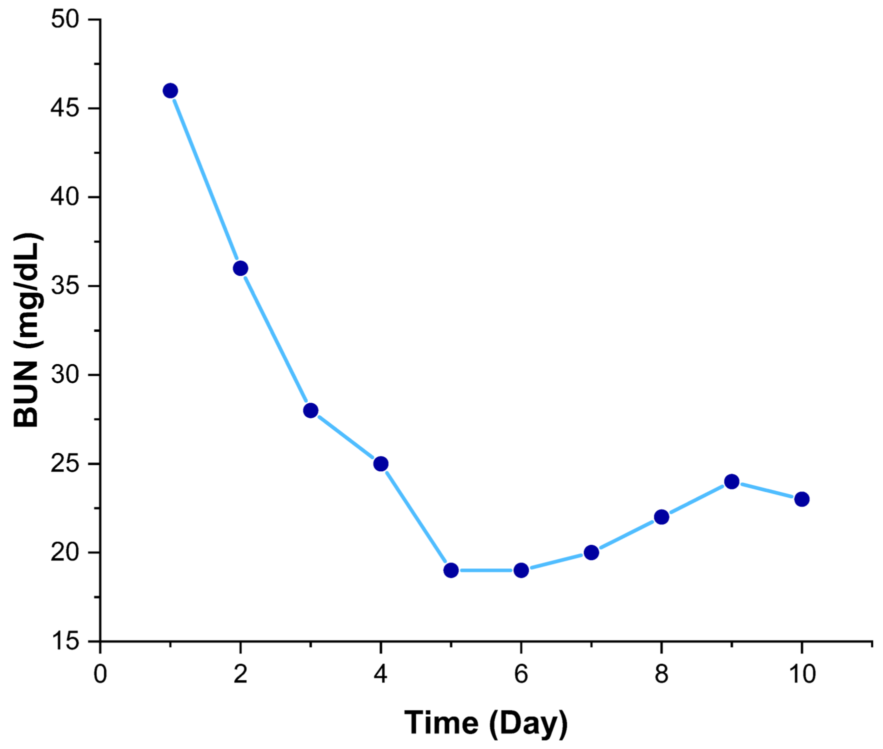

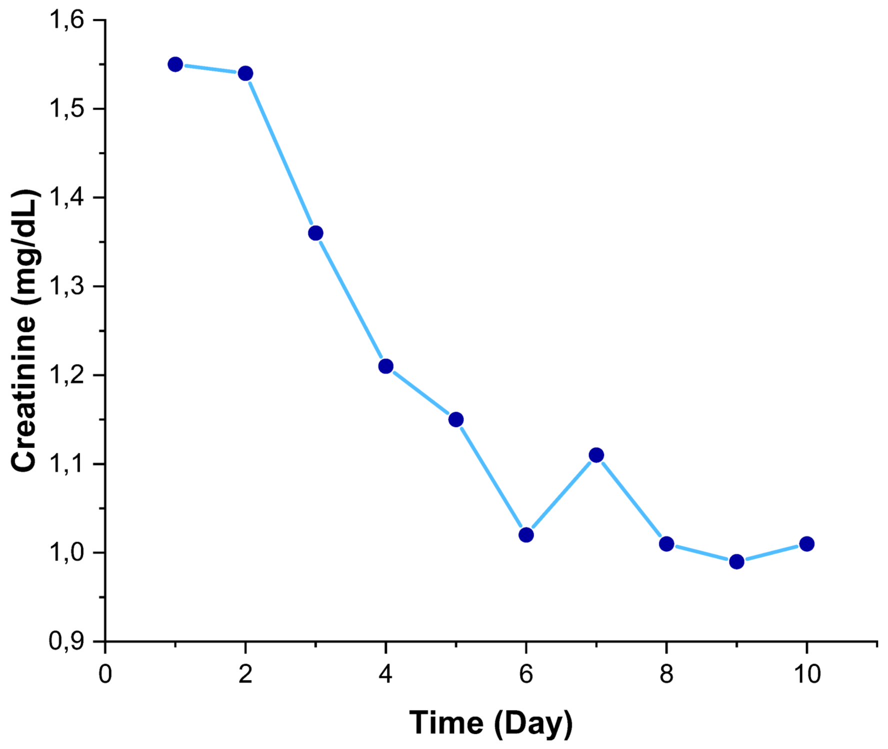

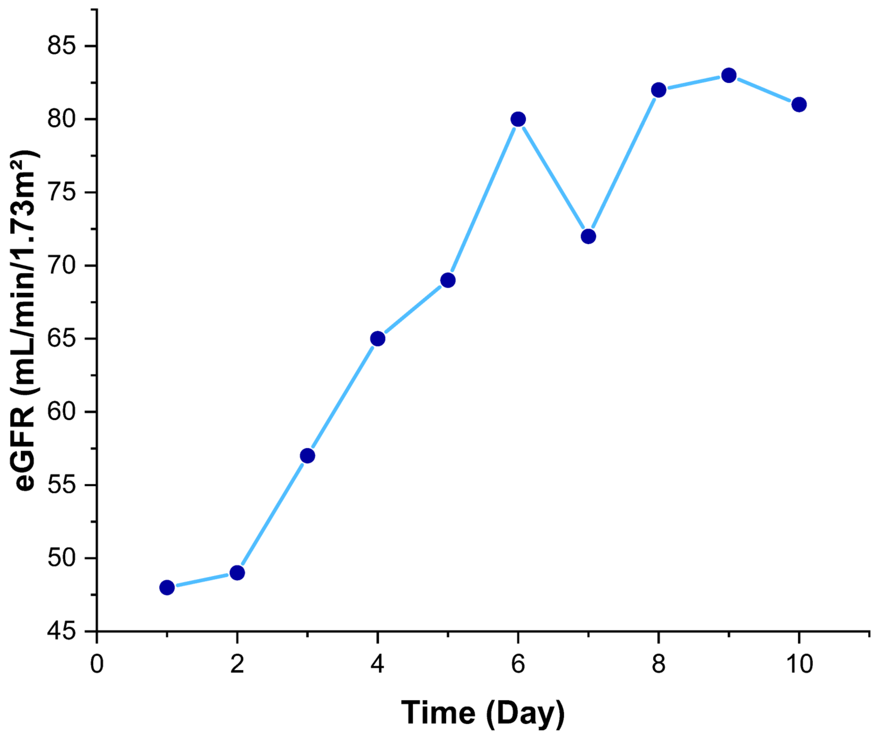

Figure S11. Patient ID: 11 demographic, clinical, and biochemical parameters. BMI: Body Mass Index, DM: Diabetes Mellitus; HT: Hypertension, CAD: Coronary Artery Disease, CKD: Chronic Kidney Disease

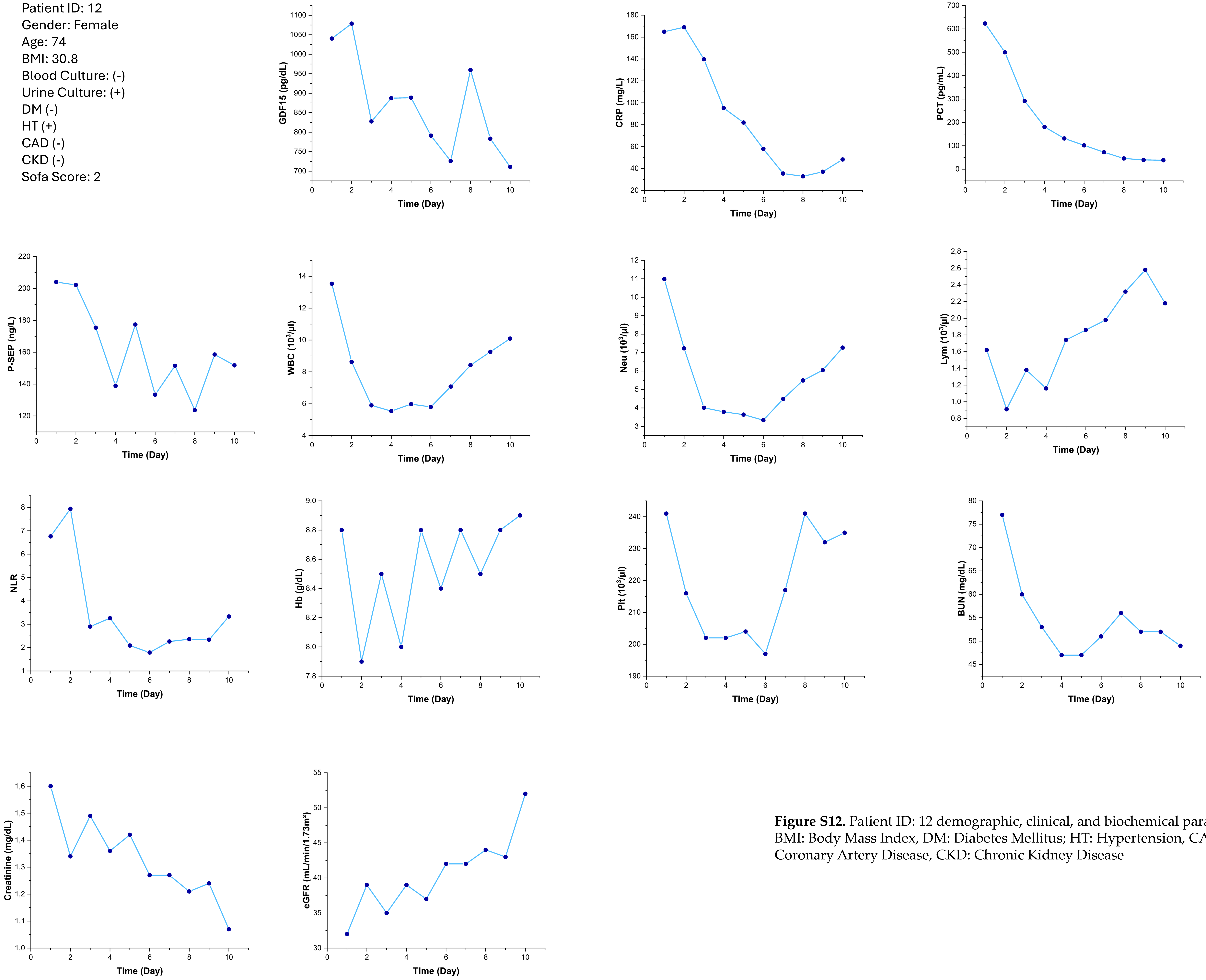

Figure S12. Patient ID: 12 demographic, clinical, and biochemical parameters. BMI: Body Mass Index, DM: Diabetes Mellitus; HT: Hypertension, CAD: Coronary Artery Disease, CKD: Chronic Kidney Disease

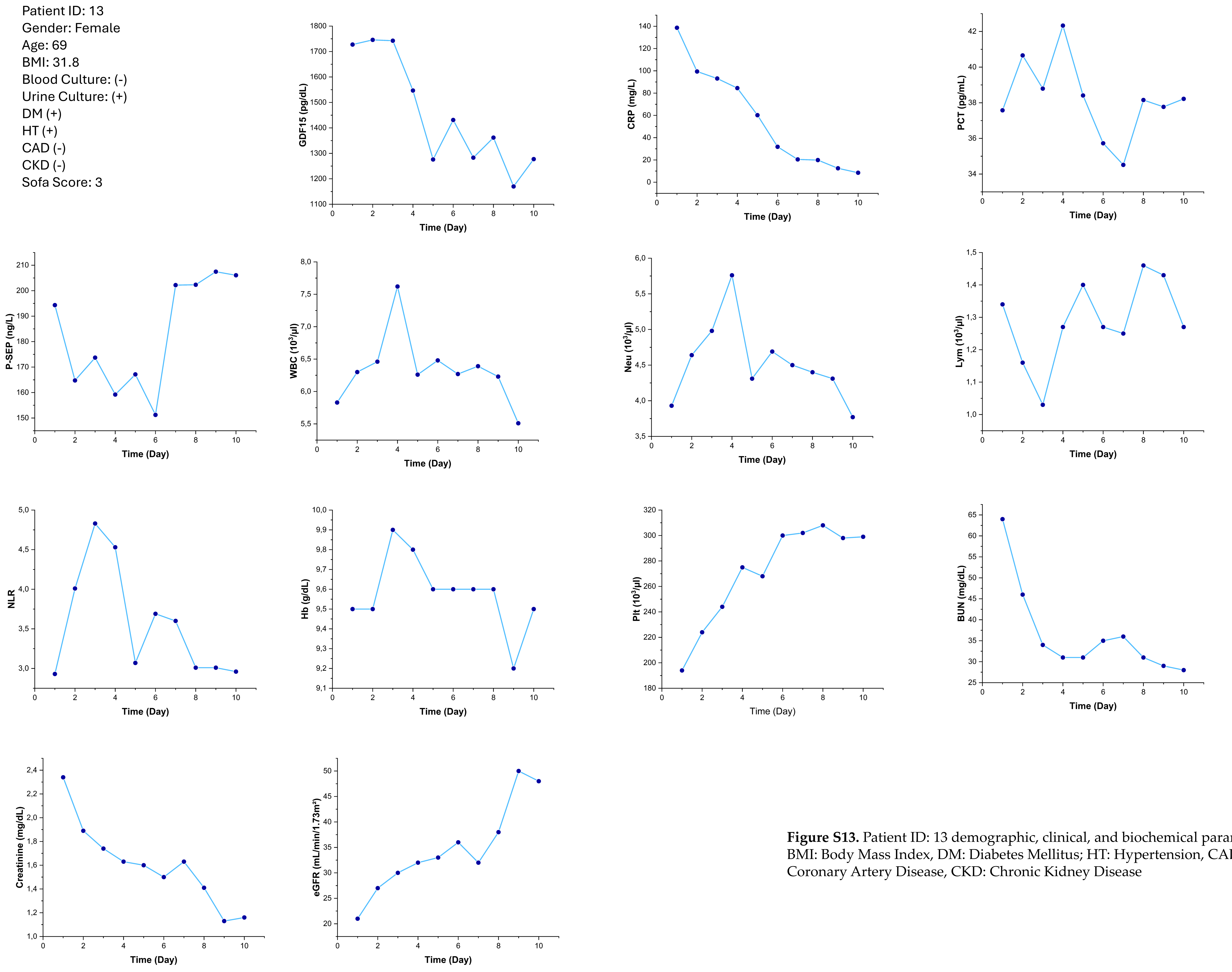

Figure S13. Patient ID: 13 demographic, clinical, and biochemical parameters. BMI: Body Mass Index, DM: Diabetes Mellitus; HT: Hypertension, CAD: Coronary Artery Disease, CKD: Chronic Kidney Disease
